# SpheroScan: a user-friendly deep learning tool for spheroid image analysis

**DOI:** 10.1093/gigascience/giad082

**Published:** 2023-10-27

**Authors:** Akshay Akshay, Mitali Katoch, Masoud Abedi, Navid Shekarchizadeh, Mustafa Besic, Fiona C Burkhard, Alex Bigger-Allen, Rosalyn M Adam, Katia Monastyrskaya, Ali Hashemi Gheinani

**Affiliations:** Functional Urology Research Group, Department for BioMedical Research DBMR, University of Bern, 3008 Bern, Switzerland; Graduate School for Cellular and Biomedical Sciences, University of Bern, 3012 Bern, Switzerland; Institute of Neuropathology, Universitätsklinikum Erlangen, Friedrich-Alexander-Universität Erlangen-Nürnberg (FAU), 91054 Erlangen, Germany; Department of Medical Data Science, Leipzig University Medical Centre, 04107 Leipzig, Germany; Department of Medical Data Science, Leipzig University Medical Centre, 04107 Leipzig, Germany; Center for Scalable Data Analytics and Artificial Intelligence (ScaDS.AI) Dresden/Leipzig, 04105 Leipzig, Germany; Functional Urology Research Group, Department for BioMedical Research DBMR, University of Bern, 3008 Bern, Switzerland; Department of Urology, Inselspital University Hospital, 3010 Bern, Switzerland; Functional Urology Research Group, Department for BioMedical Research DBMR, University of Bern, 3008 Bern, Switzerland; Department of Urology, Inselspital University Hospital, 3010 Bern, Switzerland; Biological & Biomedical Sciences Program, Division of Medical Sciences, Harvard Medical School, 02115 Boston, MA, USA; Urological Diseases Research Center, Boston Children's Hospital, Boston, MA, USA; Department of Surgery, Harvard Medical School, Boston, MA, 02115, USA; Broad Institute of MIT and Harvard, Cambridge, MA, 02142, USA; Urological Diseases Research Center, Boston Children's Hospital, Boston, MA, USA; Department of Surgery, Harvard Medical School, Boston, MA, 02115, USA; Broad Institute of MIT and Harvard, Cambridge, MA, 02142, USA; Functional Urology Research Group, Department for BioMedical Research DBMR, University of Bern, 3008 Bern, Switzerland; Department of Urology, Inselspital University Hospital, 3010 Bern, Switzerland; Functional Urology Research Group, Department for BioMedical Research DBMR, University of Bern, 3008 Bern, Switzerland; Department of Urology, Inselspital University Hospital, 3010 Bern, Switzerland; Urological Diseases Research Center, Boston Children's Hospital, Boston, MA, USA; Department of Surgery, Harvard Medical School, Boston, MA, 02115, USA; Broad Institute of MIT and Harvard, Cambridge, MA, 02142, USA

**Keywords:** 3D spheroids, deep learning, image segmentation, high-throughput screening, Image analysis, Mask R-CNN

## Abstract

**Background:**

In recent years, 3-dimensional (3D) spheroid models have become increasingly popular in scientific research as they provide a more physiologically relevant microenvironment that mimics *in vivo* conditions. The use of 3D spheroid assays has proven to be advantageous as it offers a better understanding of the cellular behavior, drug efficacy, and toxicity as compared to traditional 2-dimensional cell culture methods. However, the use of 3D spheroid assays is impeded by the absence of automated and user-friendly tools for spheroid image analysis, which adversely affects the reproducibility and throughput of these assays.

**Results:**

To address these issues, we have developed a fully automated, web-based tool called SpheroScan, which uses the deep learning framework called Mask Regions with Convolutional Neural Networks (R-CNN) for image detection and segmentation. To develop a deep learning model that could be applied to spheroid images from a range of experimental conditions, we trained the model using spheroid images captured using IncuCyte Live-Cell Analysis System and a conventional microscope. Performance evaluation of the trained model using validation and test datasets shows promising results.

**Conclusion:**

SpheroScan allows for easy analysis of large numbers of images and provides interactive visualization features for a more in-depth understanding of the data. Our tool represents a significant advancement in the analysis of spheroid images and will facilitate the widespread adoption of 3D spheroid models in scientific research. The source code and a detailed tutorial for SpheroScan are available at https://github.com/FunctionalUrology/SpheroScan.

Key points

- A deep learning model was trained to detect and segment spheroids in images from microscopes and IncuCytes.
- The model performed well on both types of images, with the total loss decreasing significantly during the training process.
- A web tool called SpheroScan was developed to facilitate the analysis of spheroid images, which includes prediction and visualization modules.
- SpheroScan is efficient and scalable, making it possible to handle large datasets with ease.
- SpheroScan is user-friendly and accessible to researchers, making it a valuable resource for the analysis of spheroid image data.

## Introduction

Two-dimensional (2D) cell culture models have long been a key component of biomedical research, but they often do not accurately replicate the *in vivo* environment [1]. In recent years, there has been an increasing realization that 3-dimensional (3D) cell cultures, such as 3D spheroid models, are better able to mimic the *in vivo* environment. Moreover, the 3D cell cultures provide more clinically relevant insights into cellular behavior and responses [2, 3]. The 3D spheroid models, in particular, have become increasingly popular due to their ability to re-create the complex microenvironment found *in vivo*. This has made them a valuable tool for studying a variety of biological processes and diseases.

Tumor spheroids are widely used for testing anticancer medications [4]. They present a compromise between the cell accessibility of adherent cultures and the 3-dimensionality of animal models. Spheroids retain more biological tumor features and reproduce the intratumor environment, which is an important feature when selecting an effective treatment strategy. Most of the spheroid-based assays use the overall size and/or cell survival as a readout [5]. Thereby, a quick and easy tool for spheroid size estimation would be advantageous for such applications.

Another important area of research that is dependent on the spheroid size evaluation is the collagen gel contraction assay (CGCA) method [6]. CGCA is a widely used *in vitro* model for studying the interactions between cells and 3D extracellular matrices. These assays help understand matrix remodeling during fibrosis and wound healing. CGCA is a competent tool to evaluate the contractility of myofibroblasts harvested from fibrotic tissues. The advent of aqueous 2-phase printing of cell-containing contractile collagen microgels has further advanced the CGCA technology [7]. Recently, the printing of the microscale cell-laden collagen gels has been combined with live-cell imaging and automated image analysis to study the kinetics of cell-mediated contraction of the collagen matrix [8]. The image analysis method utilizes a plugin for FIJI, built around Waikato Environment for Knowledge Analysis (WEKA) segmentation.

Despite the advantages of 3D spheroid models over 2D cell cultures, the lack of fully automated and user-friendly tools for analyzing spheroid images has been a major challenge, hindering widespread adoption and making high-throughput analysis difficult. Spheroid detection in an image is a crucial and challenging part of 3D spheroid assays. Several tools [9–15] have been previously developed for spheroid image analysis that utilize traditional object detection methods, such as thresholding (using algorithms like watershed [16], Otsu [17], Yen [18]), which involve setting a threshold value for the intensity of pixels and identifying all pixels above that value as part of a spheroid. Other techniques include shape-based detections (using circular/ellipse Hough transform algorithms [19], active contours models [20]) that identify spheroids based on their shape.

Unfortunately, these methods prove ineffective in adapting to a wide range of experimental conditions (Supplementary Fig. S6). The reason behind this limitation lies in the inherent variability observed in the images of spheroids captured during the assay. This variability arises from several factors, including lighting conditions, the composition of the medium, the quantity of cells utilized, treatment type, presence of debris, and variations in plate shapes, among others. Therefore, these methods require extensive fine-tuning to analyze images from each experiment and sometimes even for each specific image, which is a tedious and time-consuming task.

In recent years, the use of deep learning techniques for object detection and segmentation has significantly increased [21–25]. This rise is attributed to their ability to effectively learn from limited-size datasets and adapt to diverse imaging conditions without the need for excessive fine-tuning. Following the trend, several tools and workflows [26–31] have been developed that utilize deep learning for automatic spheroid detection in images. However, all of them require a moderate to advanced level of computational and programming skills to use. Consequently, many researchers with domain expertise are unable to utilize them easily. Additionally, none of these tools provide visualization features to allow for efficient downstream analysis of spheroid data (Supplementary Table S1). This is a significant drawback, as visualizing data can greatly aid in the interpretation and understanding of results.

**Figure 1:**
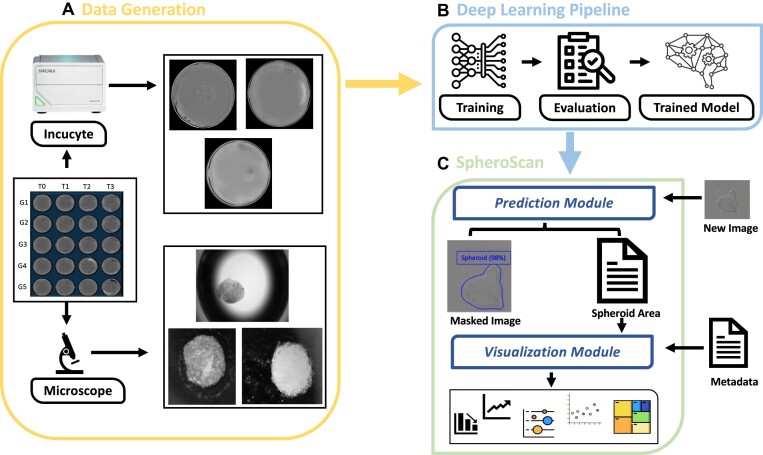
Graphical abstract. (A) Data acquisition. We used IncuCyte and microscope platforms to generate spheroid images for the training and evaluation of deep learning model. (B) Deep learning (DL) pipeline. Two models were trained using IncuCyte and microscope image datasets. These models were then evaluated on validation and test datasets. (C) SpheroScan consists of 2 submodules: prediction and visualization. The prediction module applies the trained deep learning models to mask the input spheroid images, producing a CSV file with the area and intensity of each detected spheroid as output. The visualization module enables the user to analyze the output from the prediction module by providing various plots and statistical analyses.

To address these challenges, we have developed a fully automated, user-friendly web-based tool called SpheroScan for spheroid detection and interactive visualization of spheroid data using multiple publication-ready plots. Our tool is designed to be accessible to researchers regardless of their computational skills and aims to make the process of analyzing spheroid images as simple and straightforward as possible. We have employed a state-of-the-art deep learning model called Mask R-CNN (Region-based Convolutional Neural Network) for image detection and segmentation. This model has proven to be highly effective in image analysis tasks and allows our tool to accurately detect and segment spheroids in images. With our tool, researchers can easily and quickly analyze large numbers of spheroid images and can use the interactive visualization features to gain a deeper understanding of their data (Fig. 1).

## Results and Discussion

### Training and evaluating the performance of deep learning model

**Figure 2:**
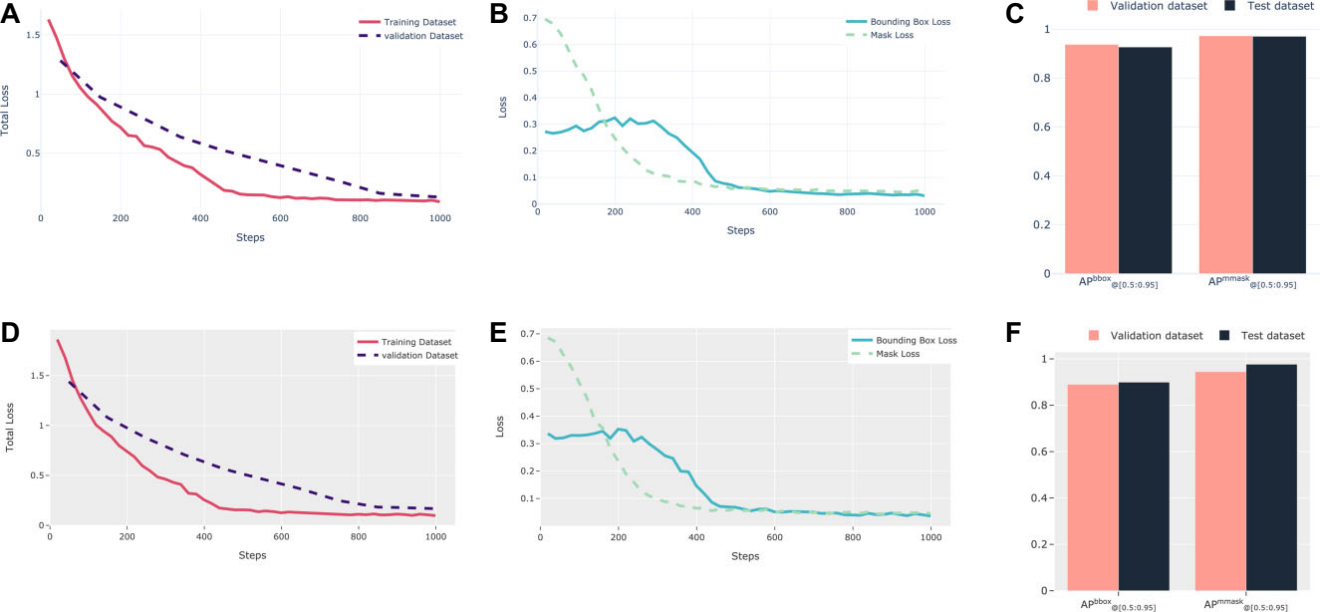
Results of the deep learning model's performance. The total loss for both training and validation datasets of IncuCyte (A) and microscope (D) images. The bounding box loss and mask loss for the training dataset of IncuCyte (B) and microscope (E) images. The APbbox_@[0.5:0.95]_ and APmmask_@[0.5:0.95]_ for the validation and test datasets of IncuCyte (C) and microscope (F) images. The APbbox_@[0.5:0.95]_ represents the average precision for bounding boxes, and the APmmask_@[0.5:0.95]_ represents the average precision for segmentation masks in the range of 0.5 to 0.95.

Figure 2 presents the performance of the trained deep learning (DL) model on the training, validation, and testing datasets for microscope and IncuCyte images. The results show that the DL model was able to effectively learn and improve its performance over the course of training for both types of images. In particular, for IncuCyte images, the total loss at baseline was 1.6 for the training data and 1.3 for the validation data. However, in the last epoch, the total loss reached its minimum values of 0.09 and 0.13 for the training and validation data, respectively (Fig. 2A). This represents a significant improvement in performance. Similarly, the bounding box and mask loss started at relatively high values of 0.3 and 0.7, respectively, but decreased to their minimum values of 0.03 and 0.04 in the last epoch (Fig. 2B). The model also performed well on the training and validation datasets for microscope images, with the total loss decreasing from 1.8 and 1.4 to 0.09 and 0.16 at the last epoch, respectively (Fig. 2D). The bounding box and mask losses for the microscope dataset were also low, 0.036 and 0.045, respectively, at the last epoch (Fig. 2E). Overall, these results demonstrate the robustness and effectiveness of the DL model in accurately detecting and segmenting spheroids in images from both microscopes and IncuCytes.

To evaluate the performance of the trained model in segmenting spheroids, we calculated the average precision (AP) metric for bounding boxes and segmentation masks in the range of 0.5 to 0.95. Throughout the text, APbbox_@[0.5:0.95]_ represents the AP for bounding boxes, and APmmask_@[0.5:0.95]_ represents the AP for segmentation masks. In general, the trained models showed similar performance on the test and validation datasets. The values for APbbox_@[0.5:0.95]_ and APmmask_@[0.5:0.95]_ were 0.937 and 0.972, respectively, for the validation data and 0.927 and 0.97, respectively, for the test data of IncuCyte images (Fig. 2C). The model's performance on the validation and test datasets for microscopic images was also strong, with scores of 0.89 and 0.944 for APbbox_@[0.5:0.95]_ and APmmask_@[0.5:0.95]_, respectively, on the validation data and scores of 0.899 and 0.977, respectively, on the test data (Fig. 2F).

Furthermore, we assessed the applicability of SpheroScan in analyzing spheroid images generated by external users using different imaging platforms, diverse cell types, growth mediums, and various lighting conditions. To achieve this objective, we employed SpheroScan to mask spheroids in multiple image datasets obtained from previous studies (Supplementary Table S2). In total, we utilized 6 distinct datasets [10, 27, 32–34], including 4 fluorescence microscopy datasets (including multichannel) and 2 brightfield microscopy datasets (Supplementary Fig. S7). The results indicate that SpheroScan effectively detected spheroids in all images from the tested datasets, affirming its adaptability and applicability to external datasets (Supplementary Table S2). Nevertheless, the Nürnberg, Elina et al. dataset posed a challenge for SpheroScan as it struggled to identify spheroids in 8 out of 48 images. The difficulty arose from a limited number of cells stained with anti-KI67, resulting in the formation of hollow spheroid-like structure.

### SpheroScan characteristics

We have developed an open-source web tool called SpheroScan to facilitate the analysis of spheroid images. This user-friendly, interactive tool is designed to streamline the process of spheroid segmentation, area calculation, and downstream analysis of spheroid image data. Furthermore, it helps to standardize and accelerate the analysis of spheroid assay results. SpheroScan consists of 2 main modules: prediction and visualization. The prediction module uses previously trained DL models to detect the spheroid in the input images; accordingly, a CSV file is generated with the area, circularity, and intensity of each detected spheroid (Supplementary Fig. S1A). The visualization module allows the user to analyze the results of the prediction module through various types of plots and statistical analyses (Supplementary Fig. S1B). The plots generated by the visualization module are ready for publication and can be saved as high-quality images in PNG format. Overall, SpheroScan is a powerful and user-friendly tool that greatly simplifies and enhances the analysis of spheroid image data (Supplementary Figs. S2–S4).

**Figure 3:**
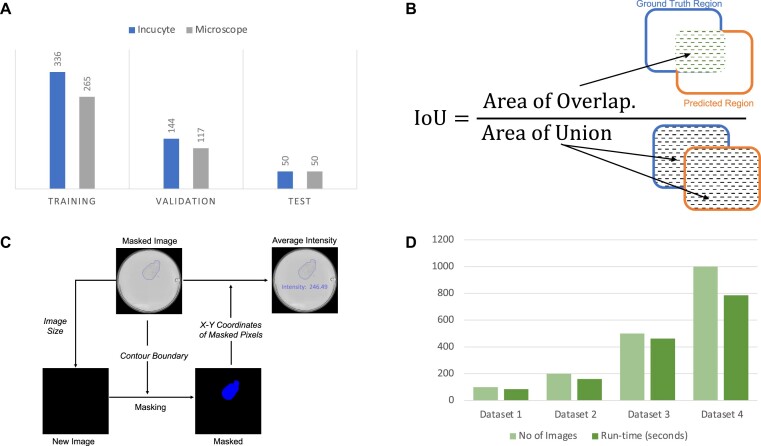
(A) Datasets. Training, validation, and test datasets size for IncuCyte and microscope models. (B) Intersection over union (IoU) metric. The IoU metric is a measure of the overlap between 2 bounding boxes or masks. It is calculated by dividing the overlap area between the predicted and ground-truth regions by the total area of both regions combined. (C) Spheroid intensity calculation. To determine the intensity of the spheroid image, a new image with the same shape and number of pixels as the original is created, but with all pixels set to zero intensity. The predicted contour boundary of the spheroid or spheroids is applied to this new image, and all pixels inside the boundary are set to 255 intensity. The x and y coordinates of each pixel in the new image with a value of 255 are then extracted. The average pixel intensity value for all points within the contour boundary is then calculated using Python's OpenCV module. (D) Runtime analysis. The runtime complexity of the prediction module was analyzed using 4 different image datasets of varying sizes. The results showed that it takes less than a second to mask an image, and the runtime complexity of the prediction module is linear. This means that the time required to process an image increases in proportion to the number of images being processed.

The runtime complexity of the prediction module is linear, meaning that it scales in proportion to the size of the input data. This is an important property because it means that the prediction module will be efficient and scalable, even when processing large datasets. To confirm the linear runtime complexity of the prediction module, we tested it on 4 different image datasets with various numbers of images. The results of these tests showed that the prediction module consistently had a linear runtime, taking less than 1 second to mask a single image (Fig. 3D). This demonstrates that the prediction module is highly efficient and capable of handling large datasets with ease. We evaluated the runtime performance on a Red Hat server with 16 central processing unit cores and 64 GB of RAM.

## Limitations and Considerations

As with any technology, there are limitations and considerations to keep in mind when using the SpheroScan system. First, it is important to note that this developed tool is primarily designed for use with the spheroid images from IncuCyte and microscope platforms. Additionally, when analyzing images that contain more than 1 spheroid, the performance of the SpheroScan system may decrease. Therefore, it is important to carefully consider the experimental design and imaging conditions to ensure optimal performance and accurate results. The authors aim to expand the training dataset with a diverse range of external images from various experimental environments and platforms in the future to improve and advance the utility of SpheroScan.

Furthermore, in the current version, the tool provides a limited set of parameters—namely, the area, circularity, and brightfield average intensity, to describe the spheroids. Although these parameters are informative and relevant for certain assays, additional parameters, such as volume estimation and cell count estimation, may be required for a more comprehensive characterization. As we continue to enhance the tool, we are actively considering incorporating derived parameters to enhance its applicability across a broader range of experimental scenarios.

Besides that, we encountered several instances where SpheroScan faced difficulties in accurately masking spheroids in images (Supplementary Fig. S8). For example, in Supplementary Fig. S8A, there was a spheroid with a hollow, spheroid-like structure formed from a limited number of labeled cells, but unfortunately, SpheroScan failed to identify it. Moreover, we noticed challenges with masking spheroid images containing debris and irregular shapes. In such situations, SpheroScan occasionally misidentified some debris as spheroids (Supplementary Fig. S8B, S8D, and S8E). However, we found that most of these challenges could be mitigated by adjusting the prediction threshold (Supplementary Fig. S8C and S8F). These challenging scenarios indicate that SpheroScan's performance may be influenced by specific image characteristics, such as the complexity of spheroid structures and the presence of debris. Generally, while the SpheroScan system offers many advantages for high-throughput spheroid analysis, it is important to be aware of its limitations and take steps to address them as needed.

## Conclusion

The development of the web-based tool SpheroScan represents a significant advancement in the analysis of 3D spheroid images. Using the state-of-the-art DL techniques, our tool accurately detects and segments spheroids in images, making it easy for researchers to analyze large numbers of spheroid images. Additionally, our tool is user-friendly and accessible to researchers regardless of their computational skills, making it a valuable resource for the scientific community. The interactive visualization features provided by our tool also allow for a more in-depth understanding of spheroid data, which will further facilitate the widespread adoption of 3D spheroid models in research. Overall, SpheroScan represents a valuable tool for researchers working with 3D spheroid models and will help to advance the use of these models in scientific research.

## Materials and Methods

### Implementation

SpheroScan (RRID:SCR_023886) was developed using the Plotly Dash [35] library in Python (version 3.10.6), and all the plots were made using Plotly. Pandas library [36, 37] was used to store and process the data.

### Spheroid image acquisition

In this study, our goal was to create a generalized DL model that can be used for spheroid images from various experimental setups or laboratory environments. To this end, we applied the aqueous 2-phase solution method to embed the cells of interest into collagen matrix spheroids. To estimate the cell-driven contraction of the collagen matrix, we collected spheroid images from different treatment conditions and time points, using both bladder smooth muscle cells (SMCs) and human embryonic kidney (HEK) cells. SMC cells were chosen for this study since they have the ability to contract, which we expected to lead to the creation of spheroids in a wide range of sizes. HEK cells, on the other hand, do not contract and were used as a negative control to ensure the accuracy of our results. The spheroids were treated with various concentrations of histamine and fetal bovine serum (FBS) and were observed at regular intervals to track their response to these treatments.

To generate the image datasets needed for a DL model, we performed a spheroid gel contraction assay using 5,000 SMC or HEK cells per collagen spheroid. After the collagen droplet polymerized, the medium was changed and plates were transferred to an IncuCyte Live-Cell Analysis System, which acquired images of the spheroids every hour for 24 hours. Alternatively, we used a ZEISS Axio Vert.A1 Inverted Microscope and manually acquired images of the spheroids at selected time points. By using both methods, we were able to capture a wide range of spheroid images and to create a robust dataset for our DL model.

A total of 480 images were obtained from the IncuCyte system, and these were randomly divided into a training dataset of 336 images (70%) and a validation dataset of 144 images (30%). An additional test dataset of 50 images was used to evaluate the performance of the trained model. To create a model specifically for microscopic images, we gathered spheroid images from the microscope and divided them into 3 datasets: training, validation, and test. The training dataset included 265 images, the validation dataset included 117 images, and the test dataset included 50 images (Fig. 3A). To test the robustness of the trained model, the spheroids in the test dataset were treated differently from those in the training and validation datasets. The medium used here was smooth muscle cell medium and Dulbecco's Modified Eagle Medium with 0.5% and 1% FBS.

In the following step, an experienced researcher in the spheroid assay manually annotated the images from IncuCyte and microscopes using the VGG Image Annotator [38].

### Deep learning framework

For spheroid detection and segmentation, we used a state-of-the-art DL model called Mask R-CNN and an open-source Python [39] library called Detectron2 [40]. Mask R-CNN is a method for solving the problem of instance segmentation, which involves both object detection and semantic segmentation. Object detection is the process of identifying and classifying multiple objects within an image, while semantic segmentation involves understanding the image at the pixel level to distinguish individual objects within the image. In order to perform these tasks, Mask R-CNN first uses a deep convolutional neural network (CNN) to process the input image and to generate a set of feature maps. These feature maps are then used as input for the next step in the process.

Mask R-CNN performs object detection in 2 stages. First, it uses a region proposal network (RPN) module to identify regions of interest (ROIs) within the image. ROIs are defined as bounding boxes with a high probability of containing objects. In the second stage, Mask R-CNN uses an ROI classifier and bounding box regressor module to classify the objects within the ROIs and to determine their bounding boxes. Both the RPN and ROI classifier and bounding box regressor modules are implemented as CNNs.

For semantic segmentation, Mask R-CNN uses a fully convolutional network (FCN) called the mask segmentation module to predict masks for each ROI determined in the object detection phase. This allows Mask R-CNN to accurately identify and distinguish individual objects within the image and segment them from the background. Overall, the combination of object detection and semantic segmentation allows Mask R-CNN to achieve highly accurate and detailed instance segmentation results (Supplementary Fig. S5).

In this study, we used the Mask R-CNN model for instance segmentation and tuned several of its parameters to fit the specific problem and the dataset we were working with. The backbone of the model was a ResNet-50 feature pyramid network, and we initialized the model with weights from a pretrained COCO instance segmentation model. The batch size for training was set to 4, and the base learning rate was set to 0.00025. The RoIHead batch size was 256, and we used a single output class (for spheroids). We trained the model for a total of 1,000 iterations. In addition to these specified parameters, we used the default values for all other parameters of the Mask R-CNN model.

### Evaluation metrics

To evaluate the performance of the trained models on spheroid segmentation, we used the AP or mean average precision (mAP) metric. mAP is a commonly used evaluation metric in computer vision for measuring the accuracy of instance segmentation and object detection models. Many of the state-of-the-art object detection algorithms, such as Faster R-CNN [41], Mask R-CNN [42], MobileNet SSD [43], and YOLO [44], as well as benchmark challenges such as PASCAL VOC [45], use AP to evaluate their models. Calculation of AP is dependent on the following metrics:

**Precision:** It is defined as the fraction of true instances among all predicted instances and is calculated using the following formula:


\begin{eqnarray*}
{\mathrm{Precision}} = \ \frac{{TP}}{{TP + FP}}
\end{eqnarray*}

**Recall**: It is a metric that represents the fraction of retrieved instances among all relevant instances and is calculated as follows:


\begin{eqnarray*}
{\mathrm{Recall}} = \ \frac{{TP}}{{TP + FN}}
\end{eqnarray*}

**IoU:** The IoU is a metric that measures the overlap between 2 bounding boxes or masks. It is commonly used to evaluate the accuracy of object detection and instance segmentation models. The IoU value ranges from 0 to 1, with a value of 1 indicating a completely accurate prediction. To calculate the IoU, the overlap between the predicted and ground-truth regions is first determined and divided by the total area of both regions. The IoU is a useful metric because it allows for comparing predictions with different shapes and sizes, as it considers the area of both the predicted and ground truth regions (Fig. 3B).

**AP:** The AP is a metric used to evaluate the performance of object detection and instance segmentation models. It is calculated as the area under the precision–recall curve, which plots the precision (the proportion of true-positive detections among all positive detections) against the recall (the proportion of true-positive detections among all ground-truth objects) of a model. AP ranges from 0 to 1, with a higher value indicating better performance. A higher AP value indicates that the model can achieve both high precision and high recall, making it a useful metric for evaluating the overall performance of a model. AP can be calculated for a specific IoU threshold as follows:


\begin{eqnarray*}
{\mathrm{AP}} = \mathop \smallint \limits_0^1 {\mathrm{Precision\ d}}\left( {{\mathrm{Recall}}} \right) \end{eqnarray*}

Often, AP is used as the average over multiple IoU thresholds, and it is calculated as follows:


\begin{eqnarray*}
{\mathrm{mAP}} = \ \frac{1}{n}\mathop \sum \limits_{k = 1}^{k = n} A{P}_k
\end{eqnarray*}

where,


\begin{eqnarray*}
A{P}_k{\mathrm{\ }} &=& {\mathrm{\ AP\ at\ }}k^{\mathrm{th}} {\mathrm{IoU\ threshold}};\\ {\mathrm{\ }}n &=& {\mathrm{\ Number\ of\ IoU\ thresholds\ under\ consideration}}. \end{eqnarray*}

In the following, AP_@0.75_ represents AP at IoU threshold 0.75 and AP_@[0.5:0.95]_ represents the average AP over 10 IoU thresholds (from 0.5 to 0.95 with a step size of 0.05).

### Area and intensity calculation

After performing object detection and instance segmentation on an image, we can use the predicted contour boundary of each spheroid to calculate its area, circularity, and intensity. To calculate the area of a spheroid, we use Python's OpenCV library to count the number of pixels within the contour boundary. This gives us the total area of the spheroid in pixels. To calculate the intensity of the spheroid, we follow a similar process. First, we create a new image with the same shape and number of pixels as the original, but with a default intensity of zero. This image is then masked with the predicted contour boundary of the spheroid, setting all pixels within the boundary to a value of 255. We then extract the x and y coordinates of all pixels with a value of 255, which correspond to the pixels within the contour boundary of the spheroid in the original image. Finally, we use OpenCV to calculate the average intensity of these pixels, which gives us the intensity value for the spheroid. This process allows us to accurately measure the area and intensity of each spheroid in an image (Fig. 3C).

## Supplementary Material

giad082_GIGA-D-23-00131_Original_Submission

giad082_GIGA-D-23-00131_Revision_1

giad082_Response_to_Reviewer_Comments_Original_Submission

giad082_Reviewer_1_Report_Original_Submission Kevin TrÃ¶ndle -- 6/16/2023 Reviewed

giad082_Reviewer_1_Report_Revision_1 Kevin TrÃ¶ndle -- 8/18/2023 Reviewed

giad082_Reviewer_2_Report_Original_Submission Francesco Pampaloni -- 7/16/2023 Reviewed

giad082_Reviewer_2_Report_Revision_1 Francesco Pampaloni -- 8/16/2023 Reviewed

giad082_Supplemental_Files

## Data Availability

The source code, example input data, and a detailed tutorial for SpheroScan are available at GitHub [46]. All supporting data, which include images used for training, validation, and testing [47], as well as the trained model weights [48], are available at Zenodo. Additionally, spheroid images from the external datasets that have been used to evaluate the applicability of SpheroScan, along with the corresponding masked images, are also available at Zenodo [49]. An archival copy of the SpheroScan code is available via the *GigaScience* database GigaDB [50].

## Additional Files

**Supplementary Table S1**. Comparison of features between SpheroScan and other similar deep learning–based tools for automatic spheroid detection. *No information provided in the manuscript. GUI = Graphical User Interface.

**Supplementary Table S2**. List of external datasets used to evaluate the performance of SpheroScan on an unseen dataset obtained from various experiments, studies, and conditions.

**Supplementary Fig. S1**. SpheroScan Graphical User Interface. (A) Prediction module. The prediction module applies trained DL models to identify and mask spheroid images. It requires a zipped folder of images, platform type, and prediction threshold as input and generates masked images and a CSV file containing the area and intensity data of the identified spheroids as output. (B) Visualization module. The visualization module creates plots and performs statistical analysis using the output file from the prediction module and a metadata file that contains information about the study design. It offers various types of plots and allows users to customize the plot options, such as plot type and color palette. Users can export plots in high-resolution PNG format.

**Supplementary Fig. S2**. SpheroScan plot gallery. (A) Bar plot. (B) Bar plot with significance level. A bar plot with significance level is a visual representation of data where the level of significance is indicated by stars. Three asterisks (***) indicate a *P* value of less than 0.001, while "ns" represents a *P* value of 0.05 or greater. The less asterisks, the lower the significance level.

**Supplementary Fig. S3**. SpheroScan plot gallery. (A) Bubble plot. A bubble plot is a type of scatterplot where the size of the bubbles represents the mean spheroid area for a certain group. The y-axis displays the relative area or contraction of the spheroid, calculated with respect to a baseline group. (B) Line plot.

**Supplementary Fig. S4**. SpheroScan plot gallery. (A) Treemap. A treemap is a method of displaying hierarchical data in which nested rectangles are used to represent different groups. The outer rectangles represent the top-level groups, while the inner rectangles represent subgroups. The size and color of each rectangle in the treemap indicate the mean spheroid areas or intensity of the corresponding group. (B) Scatterplot.

**Supplementary Fig. S5**. Mask R-CNN architecture. The Mask R-CNN model consists of 4 main modules: feature extraction, region proposal network (RPN), region of interest (ROI) classifier and bounding box regressor, and mask segmentation. The feature extraction module takes images as input and produces feature maps. The RPN module then runs on the feature maps and uses a sliding window to identify bounding boxes with a high likelihood of containing objects (ROIs). For each ROI, the ROI classifier and bounding box regressor module is used to determine the class label of the object. For semantic segmentation, the Mask R-CNN model uses a fully convolutional network (FCN) in the mask segmentation module to predict a mask for each ROI identified in the object detection phase.

**Supplementary Fig. S6**. A comparison between masking methods: thresholding approach versus SpheroScan. (A–D) Images masked using the thresholding approach in ImageJ. However, this method proves ineffective in accurately masking the spheroid due to significant contrast variations within the image. (E, F) Corresponding images masked using SpheroScan, demonstrating more accurate results.

**Supplementary Fig. S7**. Sample of spheroid images from external datasets. (A–C) Fluorescence microscopy images. (D) Fluorescence (multichannel) microscopy image. (E, F) Brightfield microscopy images.

**Supplementary Fig. S8**. Challenging scenarios encountered by SpheroScan. Image (A) contains a spheroid formed with a limited number of labeled cells exhibiting a hollow, spheroid-like structure, which was not identified by SpheroScan in this image. Images (B), (D), and (E) represent spheroid images with debris and irregular shapes, where SpheroScan mistakenly identified debris as spheroids at a prediction threshold of 0.8. To address this issue, the threshold was adjusted to 0.95 for image (B) and 0.9 for image (E), leading to correct masking, as shown in images (C) and (F), respectively. However, even after increasing the threshold, SpheroScan still failed to correctly mask the spheroid in image (D).

## Abbreviations

AP: average precision; CGCA: collagen gel contraction assay; CNN: convolutional neural network; FBS: fetal bovine serum; FCN: fully convolutional network; HEK: human embryonic kidney; mAP: mean average precision; R-CNN: Region-based Convolutional Neural Network; ROI: region of interest; RPN: region proposal network; SMC: smooth muscle cell; WEKA: Waikato Environment for Knowledge Analysis.

## Availability of Supporting Source Code and Requirements

Project name: SpheroScan

Project homepage: https://github.com/FunctionalUrology/SpheroScan

BioTool ID: spheroscan

SciCrunch ID: SpheroScan (RRID:SCR_023886)

Operating system(s): Linux or Mac

Programming language: Python 3.10.6

Other requirements: Docker, Python, Anaconda, Git

License: GNU GPL
