# Supplementary material for: SpheroScan: a user-friendly deep learning tool for spheroid image analysis
Source: Gigascience. 2023 Oct 27;12:giad082. doi: 10.1093/gigascience/giad082 (PMC10603766; doi:10.1093/gigascience/giad082)
Supplement: giad082_GIGA-D-23-00131_Revision_1 [file giad082_giga-d-23-00131_revision_1.pdf]

GigaScience

SpheroScan: A User-Friendly Deep Learning Tool for Spheroid Image Analysis

--Manuscript Draft--

**Manuscript Number:**	GIGA-D-23-00131R1
**Full Title:**	SpheroScan: A User-Friendly Deep Learning Tool for Spheroid Image Analysis
**Article Type:**	Technical Note
**Funding Information:**	Schweizerischer Nationalfonds zur Förderung der Wissenschaftlichen Forschung (310030_175773) — Dr Fiona C. Burkhard<br>Schweizerischer Nationalfonds zur Förderung der Wissenschaftlichen Forschung (212298) — Dr Fiona C. Burkhard<br>Wings for Life (WFL-AT-06/19) — Dr Katia Monastyrskaya<br>Else Kröner-Fresenius-Stiftung (2021_EKeA.33) — Not applicable<br>Sächsisches Staatsministerium für Wissenschaft und Kunst (ScaDS.AI) — Not applicable
**Abstract:**	Background<br>In recent years, three-dimensional (3D) spheroid models have become increasingly popular in scientific research as they provide a more physiologically relevant microenvironment that mimics in vivo conditions. The use of 3D spheroid assays has proven to be advantageous as it offers a better understanding of the cellular behavior, drug efficacy, and toxicity as compared to traditional two-dimensional cell culture methods. However, the use of 3D spheroid assays is impeded by the absence of automated and user-friendly tools for spheroid image analysis, which adversely affects the reproducibility and throughput of these assays.<br>Results<br>To address these issues, we have developed a fully automated, web-based tool called SpheroScan, which uses the deep learning framework called Mask Regions with Convolutional Neural Networks (R-CNN) for image detection and segmentation. To develop a deep learning model that could be applied to spheroid images from a range of experimental conditions, we trained the model using spheroid images captured using IncuCyte Live-Cell Analysis System and a conventional microscope. Performance evaluation of the trained model using validation and test datasets shows promising results.<br>Conclusion<br>SpheroScan allows for easy analysis of large numbers of images and provides interactive visualization features for a more in-depth understanding of the data. Our tool represents a significant advancement in the analysis of spheroid images and will facilitate the widespread adoption of 3D spheroid models in scientific research. The source code and a detailed tutorial for SpheroScan are available at https://github.com/FunctionalUrology/SpheroScan.
**Corresponding Author:**	Ali Hashemi Gheinani<br>Boston Children's Hospital<br>SWITZERLAND
**Corresponding Author Secondary Information:**	
**Corresponding Author's Institution:**	Boston Children's Hospital
**Corresponding Author's Secondary Institution:**	
**First Author:**	Akshay Akshay
**First Author Secondary Information:**	

Powered by Editorial Manager® and ProduXion Manager® from Aries Systems Corporation

**Order of Authors:**	Akshay Akshay
	Mitali Katoch
	Masoud Abedi
	Navid Shekarchizadeh
	Mustafa Besic
	Fiona C. Burkhard
	Alex Bigger-Allen
	Rosalyn M. Adam
	Katia Monastyrskaya
	Ali Hashemi Gheinani
**Order of Authors Secondary Information:**	

Response to Reviewers:

Dear Editors and Dear reviewers,

We would like to express our gratitude for the thoughtful review and constructive feedback provided by the reviewers for our manuscript titled "SpheroScan: A User-Friendly Deep Learning Tool for Spheroid Image Analysis" (Manuscript ID: GIGA-D-23-00131). We sincerely appreciate the time and effort invested in evaluating our work, and we are committed to addressing the reviewers' concerns to enhance the quality and impact of our research. In light of the reviewers' valuable suggestions, we have carefully considered their comments and have prepared a detailed response to each of the raised points:

##
Reply to Editor
##
Editor reports:
In particular, the reviewers would like to see additional comparisons and validation (e.g. with external data). Reviewer #2 also points out that the web tool does not seem to be functional. In addition, please register any new software application in the bio.tools and SciCrunch.org databases to receive RRID (Research Resource Identification Initiative ID) and biotoolsID identifiers, and include these in your manuscript.

Answer: Thank you for your valuable feedback. In compliance with your request, we have included these unique identifiers in the manuscript. They can be found at line 481 and 482 in main manuscript under Availability of supporting source code and requirements.
https://bio.tools/spheroscan
BioTool ID: spheroscan
SciCrunch ID: SpheroScan (RRID:SCR_023886)

##
Reply to Reviewer 1
##

Reviewer reports:
Reviewer #1: The authors present a "Technical Note" about an open-source web tool called SpheroScan. As input users could upload (large batches of) spheroid images (brightfield, 2D). The tool delivers two outputs: (1) Prediction Module: creates a file with area and intensity of detected spheroids (CSV), (2) Visualization Module: plots of the corresponding parameters (PNG). Performance was tested on 480 Incucyte images and 423 microscope images with 336 (70 %) and 265 for training, 144 (30 %) and 117 for validation, and 50 images for testing, respectively. The framework is based on Mask R-CNN and Detectron2 library. The performance was tested in the range of 0.5 to 0.95 against manual annotation (VGG Annotator). As evaluation measure they used Intersection over union (IoU), determining the overlap between the predicted and

Powered by Editorial Manager® and ProduXion Manager® from Aries Systems Corporation

ground truth regions and calculates values of Average Precision (AP) for masking: 0.937 and 0.972 (Test), 0.927 and 0.97 (Validation) as well as AP for bounding box: 0.899 and 0.977 (test) 0.89 and 0.944 (Validation). They show a linear runtime, proofed with different sized datasets (1 s / image) for masking on a 16 core CPU, 64 GB RAM machine. The tool is available on GitHub and claimed to be available as a web tool on spheroscan.onrender.com.

General evaluation:

The concept of the tool serves some important needs of 3D cell culture-based assays: automated, standardized, high-throughput image analysis. As such, it represents value added for the research field. However, it remains open how high the impact, the reproducibility, and the chances of potential application by other researchers will be. This is due to some significant limitations in accessibility (i.e. non-permanent or non-functional web tool), as well as the (potential) restriction of input data (i.e. brightfield only, not validated with external data) and the limited options for analysis of the metadata (i.e. area and intensity only). The greatest value stems from the possibility to access a web interface, which is easy to use and will ideally be equipped with additional functionalities in the future.

Answer---------> We thank the reviewer for their insightful comments and valuable feedback on our manuscript. Your comments were very helpful, and we have taken them into account in the revised version. We appreciate your time and efforts in helping to improve the quality of our work.

Regarding “the limitations in accessibility, non-permanent or non-functional web too”l: We have purchased the infrastructure and incorporated our tool into the web site that can be accessible and tested to be functional. The address is: http://spheroscan.appengine.flow.ch/. Added to github.

Regarding “the (potential) restriction of input data (i.e. brightfield only, not validated with external data) and the limited options for analysis of the metadata (i.e. area and intensity only)”:

In response to this constructive comment, the tool SpheroScan was tested for its usability in analyzing spheroid images from various sources, including different imaging platforms, cell types, growth mediums, and lighting conditions. Added to line 233-244

Comment 1 (minor):

The presented tool uses the Mask R-CNN deep-learning model in their image processing pipeline. Several tools, which perform image segmentation, are based on this or other models are well-established and already implemented in several commercial imaging devices and allow for segmentation of cell containing image areas, e.g. to determine confluency or cell migration in "wound healing assays", mainly optimized for 2D cultures, but also applicable for 2D images of 3D spheroids. The concept of automated image segmentation is thus not novel and only meets the journal's input criterion as "update or adaptation of existing" tools.

The state-of-the-art and preliminary work are not sufficiently referenced. Several similar and alternative (open-source) tools are existent and should be mentioned in the manuscript, e.g. (Lacalle et al., 2021; Piccinini et al., 2023; Trossbach et al., 2023), to give only a few examples.

Answer---------> Thank you for your valuable feedback. We appreciate your insightful comments regarding the need to provide more comprehensive references to state-of-the-art and preliminary work in our manuscript. We have have made significant improvements to the Introduction section to address this concern. Specifically, we have extended the Introduction (lines 155 -185) to include a more thorough background on the subject matter. Additionally, we have created Table S1, which provides a comparative summary of similar existing tools and their key features.

Comment 2 (major):

The authors claim to present an user-friendly open-source web tool. The python project is available on Github, and on a demo-server (https://urldefense.com/v3/__https://spheroscan.onrender.com/__;!!NZvER7FxgEiBAiR_!tgxP4EUU1Jepc_8xvUk9fyvwMViXXppwOWtTyQvxKszSAerS27mDkOkYQRG0KyEEsz6AiI9aRAU12NyAb_GvMy6F85AOrxTAQ$) where the web interface can be

accessed. Unfortunately the mentioned web tool is not functional, i.e. it is stated on the website: "This is a demonstration server and the prediction module is not available for use. To utilize the prediction functionality, please run SpheroScan on your local machine.". This is significantly limiting the applicability of the presented tool to users who are able to execute python code on their local hardware. Therefore, the demo server should either present a functional user interface (recommended), or the statement should be removed from the manuscript, which would limit the impact of the submission significantly.

Answer---------> Thank you for your feedback and bringing the issue regarding the functionality of our web tool to our attention. We apologize for any confusion caused by the current state of the demo server. We are pleased to inform you that the prediction module is now fully active on the server, and the web tool is functional as intended. Users can access and utilize the web interface without the need to run SpheroScan on their local machines. The web server can be accessed at the following URL: http://spheroscan.appengine.flow.ch/ as it is mentioned in the github.

Comment 3 (major):
The presented algorithm was trained exclusively on internal data of brightfield images from "Incucyte and microscope platforms". Furthermore, two distinct models were generated, working with either Incucyte or microscope images.
It remains unclear how the algorithm will perform on external data of prospective users. Given the fact that two distinct models had to be trained for different image sources (i.e. from two different platforms) indicates a limited robustness of the models in this regard. This is clearly a general problem of image processing algorithms, but one that will stand in the way of applicability by external users with certainly other imaging techniques. Since the web tool interface is not functional at this point, the authors will also not be able to evaluate or improve on this after publication. At least one performance test with external data, obtained from an ideally blinded user should be performed, to further elaborate on this.

Answer---------> Thank you for providing us with your valuable feedback regarding testing the applicability of SpheroScan on external datasets. Your suggestion has been taken into consideration, and we deeply appreciate your input.
To assess the applicability of SpheroScan to external data, we utilized six distinct datasets, including four fluorescence microscopy datasets (including multichannel) and two brightfield microscopy datasets, obtained from previous studies. These datasets are from various imaging platforms, cell types, growth mediums, and lighting conditions. We employed SpheroScan to mask spheroids in the images from these datasets, and the results demonstrated that SpheroScan successfully identified spheroids in all images. This validation shows that SpheroScan is adaptable and suitable for analyzing spheroid images generated by external users with different imaging techniques. Nevertheless, the Nürnberg, Elina et al. dataset posed a challenge for SpheroScan as it struggled to identify spheroids in 8 out of 48 images. The difficulty arose from a limited number of labelled cells stained with anti-KI67, resulting in the formation of hollow spheroid-like structures.
We have included the corresponding text in the updated manuscript as well. Please refer to line 233-244. Kindly refer to line 519 – 522. for the used external datasets and corresponding masked images.

Comment 4 (major):
Many assays nowadays use fluorescent labels, for example to calculate cell ratios within 3D arrangements, e.g. for cell viability or the expression of certain proteins. The authors do not state if the algorithm (or future iterations thereof) is or will be able to process multi-channel microscope images of spheroids.
This is a significant limitation of the presented work and should at least be mentioned in the corresponding section, respectively. Furthermore, a proof-of-concept test run with fluorescent images could be performed to test the algorithm performance and derive potentially necessary adaptations in future versions.

Answer---------> Thank you for providing us with your feedback regarding testing the applicability of SpheroScan to fluorescent (multichannel) datasets.

To assess the applicability of SpheroScan fluorescent (multichannel) dataset, we utilized four fluorescence microscopy datasets (including multichannel) obtained from previous studies. We employed SpheroScan to mask spheroids in the images from these datasets, and the results demonstrated that SpheroScan successfully identified spheroids in all images. This validation shows that SpheroScan is adaptable and suitable for analyzing spheroid images generated by external users with different imaging techniques. Nevertheless, the Nürnberg, Elina et al. dataset posed a challenge for SpheroScan as it struggled to identify spheroids in 8 out of 48 images. The difficulty arose from a limited number of cells stained with anti-KI67, resulting in the formation of hollow spheroid-like structures.
We have included the corresponding text in the updated manuscript as well. Please refer to line 233-244. Kindly refer to line 519 – 522.for the used external datasets and corresponding masked images.
We have also extended the limitation section to address the challenges posed by Nürnberg, Elina et al. dataset. Please refer to line 295 – 305.

Comment 5 (minor):
The output of the tool is a list of detected spheroids with corresponding area (2D) and bright field average intensity within the area. The usability of these two parameters is limited to specific assays, such as the mentioned use case to investigate collagen gel contraction assays. Several other parameters of interest could easily be derived from the metadata, such as roundness, volume estimation (assuming a spheroid shape), or even cell count estimation. This should again be mentioned in the "limitations and considerations" section.

Answer---------> We greatly appreciate your valuable feedback. In response to your comment, we have now included the roundness parameter in our tool. With the inclusion of the roundness parameter, users can gain further insights into the spheroid morphology, enhancing the applicability of our tool to a broader range of assays and experimental setups.
Moreover, we have extended the "limitations and considerations" section in the manuscript to provide a more detailed explanation regarding the limited number of parameters currently available in our tool. Please refer to line 287 – 293. As we continue to improve the tool, we will consider incorporating these additional parameters in future updates to enhance its versatility and usefulness across various experimental scenarios.

##
Reply to Reviewer 2
##

Reviewer #2: This study represents a significant contribution to the field of screening and analysis of three-dimensional cell cultures. The demand for reliable and user-friendly image processing tools to extract quantitative data from a large number of spheroids or other types of three-dimensional tissue models is substantial. The authors of this manuscript have developed a tool that aims to address this need by providing a straightforward method to extract the projected area and intensity of individual cellular spheroids imaged with bright-field microscopy. The tool is compatible with "Incucyte" microscopes or any other automated microscope capable of imaging multiple specimens, typically found in high-density multiwell plates.An admirable aspect of this work is the authors' decision to make all the code and pipeline openly available on Github. This openness allows other scientists to test and validate the code, promoting transparency and collaboration in the scientific community. However, several improvements should be made to the manuscript prior to publication.
One important aspect that the authors should address in the manuscript is the suitability, rationale, and extent of using a neural network-based segmentation approach for the specific analysis described in the manuscript—segmentation of single bright-field images of spheroids. While neural networks are anticipated to play an increasingly important role in microscopy data segmentation in the coming years, they are not a universal solution. Although there may be segmentation tasks that are challenging to accomplish with traditional approaches, where neural networks can be highly effective, other segmentation tasks can be successfully performed using conventional strategies. For example, in our research group, we were able to reliably

Powered by Editorial Manager® and ProduXion Manager® from Aries Systems Corporation

	segment densely populated bright-field images containing numerous organoids in a single field of view using a pipeline based on the ImageJ plugin MorphoLibJ (see references: https://urldefense.com/v3/__https://doi.org/10.1093/bioinformatics/btw413__;!!NZvER7FxgEiBAiR_!tgxP4EUU1Jpepc_8xvUk9fyvwMViXXppwOWtTyQvxKszSAerS27mDkOkYQRG0KyEEsz6AiI9aRAU12NyAb_GvMy6F85GbVTAsQ$ and https://urldefense.com/v3/__https://doi.org/10.1186/s12915-021-00958-w__;!!NZvER7FxgEiBAiR_!tgxP4EUU1Jpepc_8xvUk9fyvwMViXXppwOWtTyQvxKszSAerS27mDkOkYQRG0KyEEsz6AiI9aRAU12NyAb_GvMy6F87uG8QyzQ$). Therefore, it would be informative and valuable for readers if the authors compared the results obtained from the neural network with those achieved by employing simple thresholding techniques (such as Otsu or Watershed) on the same dataset, as demonstrated in a similar study (reference: https://urldefense.com/v3/__https://doi.org/10.1038/s41598-021-94217-1__;!!NZvER7FxgEiBAiR_!tgxP4EUU1Jpepc_8xvUk9fyvwMViXXppwOWtTyQvxKszSAerS27mDkOkYQRG0KyEEsz6AiI9aRAU12NyAb_GvMy6F87782indg$, Figure 5).<br><br>Answer---------> Thank you for careful review of our manuscript. Your comments were very helpful, and we have taken them into account in the revised version. We appreciate your time and efforts in helping to improve the quality of our work.<br>We have have made significant improvements to the Introduction section to address this concern. Specifically, we have extended the Introduction (lines 155 -185) to include a more thorough background on the subject matter. Furthermore, we have included a figure with example images to demonstrate potential scenarios where thresholding algorithms may fail. Please refer to figure S6<br><br>--<br>Furthermore, to address the limitations of the model, the authors should provide specific examples (preferably in the supplementary material due to space constraints) of incorrect segmentations or artifacts that arise from applying the neural network to the data. For instance, it would be beneficial to explore scenarios where spheroids are surrounded by cellular debris or when multiple spheroids are present in the field of view. These real-life situations are common and it is important to provide insights into potential challenges that may arise when the images of the spheroids are not pristine<br><br>Answer---------> Thank you for your valuable feedback. We have included a supplementary figure (figure S8) that presents some example images posing challenges to SpheroScan for masking. We have also extended the limitation section to address this concern. Please refer to line 295 – 305.
**Additional Information:**	
**Question**	**Response**
Are you submitting this manuscript to a special series or article collection?	No
**Experimental design and statistics**<br><br>Full details of the experimental design and statistical methods used should be given in the Methods section, as detailed in our Minimum Standards Reporting Checklist. Information essential to interpreting the data presented should be made available in the figure legends.<br><br>Have you included all the information requested in your manuscript?	Yes

Powered by Editorial Manager® and ProduXion Manager® from Aries Systems Corporation

**Resources**<br><br>A description of all resources used, including antibodies, cell lines, animals and software tools, with enough information to allow them to be uniquely identified, should be included in the Methods section. Authors are strongly encouraged to cite Research Resource Identifiers (RRIDs) for antibodies, model organisms and tools, where possible.<br><br>Have you included the information requested as detailed in our Minimum Standards Reporting Checklist?	Yes
**Availability of data and materials**<br><br>All datasets and code on which the conclusions of the paper rely must be either included in your submission or deposited in publicly available repositories (where available and ethically appropriate), referencing such data using a unique identifier in the references and in the "Availability of Data and Materials" section of your manuscript.<br><br>Have you have met the above requirement as detailed in our Minimum Standards Reporting Checklist?	Yes

Powered by Editorial Manager® and ProduXion Manager® from Aries Systems Corporation

Manuscript
Click here to access/download;Manuscript;SpheroScan_for_production.docx

SpheroScan: A User-Friendly Deep Learning Tool for Spheroid Image Analysis

Akshay Akshay[1,2], Mitali Katoch[3], Masoud Abedi[4], Navid Shekarchizadeh[4,6], Mustafa Besic[1,5],Fiona C. Burkhard[1,5], Alex Bigger-Allen[7,8,9,10], Rosalyn M. Adam[8,9,10], Katia Monastyrskaya[1, 5] and Ali Hashemi Gheinani[1,5,8,9,10*]

[1] Functional Urology Research Group, Department for BioMedical Research DBMR, University of Bern, Switzerland

[2] Graduate School for Cellular and Biomedical Sciences, University of Bern, Switzerland

[3] Institute of Neuropathology, Universitätsklinikum Erlangen, Friedrich-Alexander-Universität Erlangen-Nürnberg (FAU), Erlangen, Germany

[4] Department of Medical Data Science, Leipzig University Medical Centre, 04107 Leipzig, Germany

[5] Department of Urology, Inselspital University Hospital, 3010 Bern, Switzerland

[6] Center for Scalable Data Analytics and Artificial Intelligence (ScaDS.AI) Dresden/Leipzig, 04105 Leipzig, Germany

[7] Biological & Biomedical Sciences Program, Division of Medical Sciences, Harvard Medical School, Boston, MA.

[8] Urological Diseases Research Center, Boston Children's Hospital, MA, USA

[9] Harvard Medical School, Boston, Department of Surgery MA, USA

[10] Broad Institute of MIT and Harvard, Cambridge, MA, USA

ORCID iDs: Akshay Akshay [0000-0003-3186-7478]; Mitali Katoch [0000-0002-9248-6348]; Masoud Abedi [0000-0003-3986-4028]; Navid Shekarchizadeh [0000-0002-5750-7801]; Mustafa Besic []; Fiona C Burkhard [0000-0002-8271-014X]; Alex Bigger-Allen [0000-0001-6914-9135]; Rosalyn M Adam [0000-0002-0943-6236]; Katia Monastyrskaya [0000-0003-2042-1139]; Ali Hashemi Gheinani [0000-0002-9625-6259];

* Corresponding author:

Ali Hashemi Gheinani, Urological Diseases Research Center, Boston Children's Hospital, Harvard Medical School and Broad Institute of MIT and Harvard, Cambridge, MA, USA

e-mail: Ali.HashemiGheinani@childrens.harvard.edu

Keywords

- 3D spheroids
- Deep learning
- Image segmentation
- High-throughput screening
- Image analysis
- Mask R-CNN

Key Points

- A deep learning model was trained to detect and segment spheroids in images from microscopes and Incucytes.
- The model performed well on both types of images with the total loss decreasing significantly during the training process.
- A web tool called SpheroScan was developed to facilitate the analysis of spheroid images, which includes prediction and visualization modules.
- SpheroScan is efficient and scalable, making it possible to handle large datasets with ease.
- SpheroScan is user-friendly and accessible to researchers, making it a valuable resource for the analysis of spheroid image data.

Abstract

Background

In recent years, three-dimensional (3D) spheroid models have become increasingly popular in scientific research as they provide a more physiologically relevant microenvironment that mimics in vivo conditions. The use of 3D spheroid assays has proven to be advantageous as it offers a better understanding of the cellular behavior, drug efficacy, and toxicity as compared to traditional two-dimensional cell culture methods. However, the use of 3D spheroid assays is impeded by the absence of automated and user-friendly tools for spheroid image analysis, which adversely affects the reproducibility and throughput of these assays.

Results

To address these issues, we have developed a fully automated, web-based tool called SpheroScan, which uses the deep learning framework called Mask Regions with Convolutional Neural Networks (R-CNN) for image detection and segmentation. To develop a deep learning model that could be applied to spheroid images from a range of experimental conditions, we trained the model using spheroid images captured using IncuCyte Live-Cell Analysis System and a conventional microscope. Performance evaluation of the trained model using validation and test datasets shows promising results.

Conclusion

SpheroScan allows for easy analysis of large numbers of images and provides interactive visualization features for a more in-depth understanding of the data. Our tool represents a significant advancement in the analysis of spheroid images and will facilitate the widespread adoption of 3D spheroid models in scientific research. The source code and a detailed tutorial for SpheroScan are available at https://github.com/FunctionalUrology/SpheroScan.

Introduction

Two-dimensional (2D) cell culture models have long been a key component of biomedical research, but they often do not accurately replicate the in vivo environment[1]. In recent years, there has been an increasing realization that three-dimensional (3D) cell cultures, such as 3D spheroid models, are better able to mimic the in vivo environment. Moreover, the 3D cell cultures provide more clinically relevant insights into cellular behaviour and responses[2,3]. The 3D spheroid models, in particular, have become increasingly popular due to their ability to recreate the complex microenvironment found in vivo. This has made them a valuable tool for studying a variety of biological processes and diseases.

Tumour spheroids are widely used for testing anti-cancer medications[4]. They present a compromise between the cell accessibility of adherent cultures and the three-dimensionality of animal models. Spheroids retain more biological tumor features and reproduce intra-tumour environment, which is an important feature when selecting an effective treatment strategy. Most of the spheroid-based assays use the overall size and/or cell survival as a readout[5]. Thereby, a quick and easy tool for spheroid size estimation would be advantageous for such applications.

Another important area of research that is dependent on the spheroid size evaluation is the collagen gel contraction assay (CGCA) method[6]. CGCA is a widely used in vitro model for studying the interactions between cells and 3D extracellular matrices. These assays help understand matrix remodelling during fibrosis and wound healing. CGCA is a competent tool to evaluate the contractility of myofibroblasts harvested from fibrotic tissues. The advent of aqueous two-phase printing of cell-containing contractile collagen microgels has further advanced the CGCA technology[7]. Recently, the printing of the microscale cell-laden collagen gels has been combined with live cell imaging and automated image analysis to study the kinetics of cell-mediated contraction of the collagen matrix[8]. The image analysis method utilizes a plugin for FIJI, built around Waikato Environment for Knowledge Analysis (WEKA) Segmentation.

Despite the advantages of 3D spheroid models over 2D cell cultures, the lack of fully automated and user-friendly tools for analyzing spheroid images has been a major challenge, hindering widespread adoption and making high throughput analysis difficult. Spheroid detection in an image is a crucial and challenging part of 3D spheroid assays. Several tools[9–15]have been previously developed for spheroid image analysis that utilize traditional object detection methods, such as thresholding (using algorithms like watershed[16], Otsu[17], Yen[18]) that involve setting a threshold value for the intensity of pixels and identifying all pixels above that value as part of a spheroid. Other techniques include shape-based detections (using circular/ellipse Hough transform algorithms[19], active contours models[20]) that identify spheroids based on their shape.

Unfortunately, these methods prove ineffective in adapting to a wide range of experimental conditions (Figure S6). The reason behind this limitation lies in the inherent variability observed in the images of spheroids captured during the assay. This variability arises from several factors, including lighting conditions, the composition of the medium, the quantity of cells utilized, treatment type, presence of

debris, and variations in plate shapes, among others. Therefore, these methods require extensive fine-tuning to analyze images from each experiment, and sometimes even for each specific image, which is a tedious and time-consuming task.

In recent years, the use of deep learning techniques for object detection and segmentation has significantly increased[21–25]. This rise is attributed to their ability to effectively learn from limited size datasets and adapt to diverse imaging conditions without the need for excessive fine-tuning. Following the trend, a several tools and workflows[26–31] have been developed that utilize deep learning for automatic spheroid detection in images. However, all of them require a moderate to advanced level of computational and programming skills to use. Consequently, many researchers with domain expertise are unable to utilize them easily. Additionally, none of these tools provide visualization features to allow for efficient downstream analysis of spheroid data (Table S1). This is a significant drawback, as visualizing data can greatly aid in the interpretation and understanding of results.

To address these challenges, we have developed a fully automated, user-friendly web-based tool called SpheroScan for spheroid detection and interactive visualization of spheroid data using multiple publication-ready plots. Our tool is designed to be accessible to researchers regardless of their computational skills and aims to make the process of analysing spheroid images as simple and straightforward as possible. We have employed a state-of-the-art deep learning model called Mask R-CNN (Region-based Convolutional Neural Network) for image detection and segmentation. This model has proven to be highly effective in image analysis tasks and allows our tool to accurately detect and segment spheroids in images. With our tool, researchers can easily and quickly analyse large numbers of spheroid images and can use the interactive visualization features to gain a deeper understanding of their data (Figure 1).

Results and Discussion

Training and evaluating the performance of deep learning model

Figure 2 presents the performance of the trained Deep Learning (DL) model on the training, validation, and testing datasets for microscope and Incucyte images. The results show that the DL model was able to effectively learn and improve its performance over the course of training for both types of images. In particular, for Incucyte images, the total loss at baseline was 1.6 for the training data and 1.3 for the validation data. However, in the last epoch, the total loss reached its minimum values of 0.09 and 0.13 for the training and validation data, respectively (Figure 2A). This represents a significant improvement in performance. Similarly, the bounding box and mask loss started at relatively high values of 0.3 and 0.7, respectively, but decreased to their minimum values of 0.03 and 0.04 in the last epoch (Figure 2B). The model also performed well on the training and validation datasets for microscope images, with the total loss decreasing from 1.8 and 1.4 to 0.09 and 0.16 at the last epoch, respectively (Figure 2D). The bounding box and mask losses for the microscope dataset were also low, 0.036 and 0.045, respectively, at the last epoch (Figure 2E). Overall, these results demonstrate the robustness and effectiveness of the DL model in accurately detecting and segmenting spheroids in images from both microscopes and Incucytes.

To evaluate the performance of the trained model in segmenting spheroids, we calculated the Average Precision (AP) metric for bounding boxes and segmentation masks in the range of 0.5 to 0.95. Throughout the text, APbbox@$_{[0.5:0.95]}$ represents the AP for bounding boxes, and APmmask@$_{[0.5:0.95]}$ represents the AP for segmentation masks. In general, the trained models showed similar performance on the test and validation datasets. The values for APbbox@$_{[0.5:0.95]}$ and APmmask@$_{[0.5:0.95]}$ were 0.937 and 0.972, respectively, for the validation data, and 0.927 and 0.97, respectively, for the test data of Incucyte images (Figure 2C). The model's performance on the validation and test datasets for microscopic images were also strong, with scores of 0.89 and 0.944 for APbbox@$_{[0.5:0.95]}$ and APmmask@$_{[0.5:0.95]}$ respectively on the validation data, and scores of 0.899 and 0.977 respectively on the test data (Figure 2F).

Furthermore, we assessed the applicability of SpheroScan in analyzing spheroid images generated by external users using different imaging platforms, diverse cell types, growth mediums, and various lighting conditions. To achieve this objective, we employed SpheroScan to mask spheroids in multiple image datasets obtained from previous studies (Table S2). In total, we utilized six distinct datasets[10,27,32–34], including four fluorescence microscopy datasets (including multichannel) and two brightfield microscopy datasets (Figure S7). The results indicate that SpheroScan effectively detected spheroids in all images from the tested datasets, affirming its adaptability and applicability to external datasets (Table S2). Nevertheless, the Nürnberg, Elina et al. dataset posed a challenge for SpheroScan as it struggled to identify spheroids in 8 out of 48 images. The difficulty arose from a limited number of cells stained with anti-KI67, resulting in the formation of hollow spheroid-like structure.

SpheroScan characteristics

We have developed an open-source web tool called SpheroScan to facilitate the analysis of spheroid images. This user-friendly, interactive tool is designed to streamline the process of spheroid segmentation, area calculation, and downstream analysis of spheroid image data. Furthermore, it helps to standardize and accelerate the analysis of spheroid assay results. SpheroScan consists of two main modules: prediction and visualization. The prediction module uses previously trained DL models to detect the spheroid in the input images; accordingly, a CSV file is generated with the area, circularity, and intensity of each detected spheroid (Figure S1.A). The visualization module allows the user to analyse the results of the prediction module through various types of plots and statistical analyses (Figure S1.B). The plots generated by the visualization module are ready for publication and can be saved as high-quality images in PNG format. Overall, SpheroScan is a powerful and user-friendly tool that greatly simplifies and enhances the analysis of spheroid image data (Figure S2-S4).

The runtime complexity of the prediction module is linear, meaning that it scales in proportion to the size of the input data. This is an important property because it means that the prediction module will be efficient and scalable, even when processing large datasets. To confirm the linear runtime complexity of the prediction module, we tested it on four different image datasets with various numbers of images. The results of these tests showed that the prediction module consistently had a linear runtime, taking less

than one second to mask a single image (Figure 3D). This demonstrates that the prediction module is highly efficient and capable of handling large datasets with ease. We evaluated the run-time performance on a Red Hat server with 16 Central Processing Unit (CPU) cores and 64 GB of Random-Access Memory (RAM).

Limitations and considerations

As with any technology, there are limitations and considerations to keep in mind when using the SpheroScan system. First, it is important to note that this developed tool is primarily designed for use with the spheroid images from Incucyte and microscope platforms. Additionally, when analyzing images that contain more than one spheroid, the performance of the SpheroScan system may decrease. Therefore, it is important to carefully consider the experimental design and imaging conditions to ensure optimal performance and accurate results. The authors aim to expand the training dataset with a diverse range of external images from various experimental environments and platforms in the future to improve and advance the utility of SpheroScan.

Furthermore, in the current version, the tool provides a limited set of parameters, namely the area, circularity, and bright-field average intensity, to describe the spheroids. Although these parameters are informative and relevant for certain assays, additional parameters, such as volume estimation and cell count estimation, may be required for a more comprehensive characterization. As we continue to enhance the tool, we are actively considering incorporating derived parameters to enhance its applicability across a broader range of experimental scenarios.

Besides that, we encountered several instances where SpheroScan faced difficulties in accurately masking spheroids in images (Figure S8). For example, in image S8.A, there was a spheroid with a hollow, spheroid-like structure formed from a limited number of labeled cells, but unfortunately, SpheroScan failed to identify it. Moreover, we noticed challenges with masking spheroid images containing debris and irregular shapes. In such situations, SpheroScan occasionally misidentified some debris as spheroids (S8.B, S8.D, and S8.E). However, we found that most of these challenges could be mitigated by adjusting the prediction threshold (S8.C and S8.F). These challenging scenarios indicate that SpheroScan's performance may be influenced by specific image characteristics, such as the complexity of spheroid structures and the presence of debris. Generally, while the SpheroScan system offers many advantages for high-throughput spheroid analysis, it is important to be aware of its limitations and take steps to address them as needed.

Conclusion

The development of the web-based tool SpheroScan represents a significant advancement in the analysis of 3D spheroid images. Using the state-of-the-art deep learning techniques, our tool accurately detects and segments spheroids in images, making it easy for researchers to analyse large numbers of spheroid images. Additionally, our tool is user-friendly and accessible to researchers regardless of their computational skills, making it a valuable resource for the scientific community. The interactive visualization features provided by our tool also allow for a more in-depth

understanding of spheroid data, which will further facilitate the widespread adoption of 3D spheroid models in research. Overall, SpheroScan represents a valuable tool for researchers working with 3D spheroid models and will help to advance the use of these models in scientific research.

Materials and Methods

Implementation

SpheroScan (RRID:SCR_023886) was developed using Plotly Dash[35] library in Python (version 3.10.6) and all the plots were made using Plotly. Pandas library[36,37] was used to store and process the data.

Spheroid image acquisition

In this study, our goal was to create a generalized DL model that can be used for spheroid images from various experimental setups or laboratory environments. To this end, we applied the aqueous two-phase solution method to embed the cells of interest into collagen matrix spheroids. To estimate the cell-driven contraction of the collagen matrix, we collected spheroid images from different treatment conditions and time points, using both bladder Smooth Muscle Cells (SMCs) and Human Embryonic Kidney (HEK) cells. SMC cells were chosen for this study since they have the ability to contract, which we expected to lead to the creation of spheroids in a wide range of sizes. HEK cells, on the other hand, do not contract and were used as a negative control to ensure the accuracy of our results. The spheroids were treated with various concentrations of histamine and Fetal Bovine Serum (FBS) and were observed at regular intervals to track their response to these treatments.

To generate the image datasets needed for a DL model, we performed a spheroid gel contraction assay using 5000 SMC or HEK cells per collagen spheroid. After the collagen droplet polymerized, the medium was changed and plates were transferred to an Incucyte Live-Cell Analysis System, which acquired images of the spheroids every hour for 24 hours. Alternatively, we used a ZEISS Axio Vert.A1 Inverted Microscope and manually acquired images of the spheroids at selected time points. By using both methods, we were able to capture a wide range of spheroid images and to create a robust dataset for our DL model.

A total of 480 images were obtained from the Incucyte system, and these were randomly divided into a training dataset of 336 images (70%) and a validation dataset of 144 images (30%). An additional test dataset of 50 images was used to evaluate the performance of the trained model. To create a model specifically for microscopic images, we gathered spheroid images from the microscope and divided them into three datasets: training, validation, and test. The training dataset included 265 images, the validation dataset included 117 images, and the test dataset included 50 images (Figure 3A). To test the robustness of the trained model, the spheroids in the test dataset were treated differently from those in the training and validation datasets. The medium used here was smooth muscle cell medium and Dulbecco's Modified Eagle Medium (DMEM) with 0.5% and 1% FBS.

In the following step, an experienced researcher in the spheroid assay manually annotated the images from Incucyte and microscopes using the VGG Image Annotator[38].

Deep learning framework

For spheroid detection and segmentation, we used a state-of-the-art DL model called Mask R-CNN and an open-source Python[39] library called Detectron2[40]. Mask R-CNN is a method for solving the problem of instance segmentation, which involves both object detection and semantic segmentation. Object detection is the process of identifying and classifying multiple objects within an image, while semantic segmentation involves understanding the image at the pixel level to distinguish individual objects within the image. In order to perform these tasks, Mask R-CNN first uses a deep Convolutional Neural Network (CNN) to process the input image and to generate a set of feature maps. These feature maps are then used as input for the next step in the process.

Mask R-CNN performs object detection in two stages. First, it uses a Region Proposal Network (RPN) module to identify Regions of Interest (ROIs) within the image. ROIs are defined as bounding boxes with a high probability of containing objects. In the second stage, Mask R-CNN uses an ROI classifier and bounding box regressor module to classify the objects within the ROIs and to determine their bounding boxes. Both the RPN and ROI classifier and bounding box regressor modules are implemented as CNNs.

For semantic segmentation, Mask R-CNN uses a fully convolutional network (FCN) called the mask segmentation module to predict masks for each ROI determined in the object detection phase. This allows Mask R-CNN to accurately identify and distinguish individual objects within the image and segment them from the background. Overall, the combination of object detection and semantic segmentation allows Mask R-CNN to achieve highly accurate and detailed instance segmentation results (Figure S5).

In this study, we used the Mask R-CNN model for instance segmentation and tuned several of its parameters to fit the specific problem and the dataset we were working with. The backbone of the model was a ResNet-50 feature pyramid network, and we initialised the model with weights from a pre-trained COCO instance segmentation model. The batch size for training was set to 4, and the base learning rate was set to 0.00025. The RoIHead batch size was 256, and we used a single output class (for spheroids). We trained the model for a total of 1000 iterations. In addition to these specified parameters, we used the default values for all other parameters of the Mask R-CNN model.

Evaluation Metrics

To evaluate the performance of the trained models on spheroid segmentation, we used the Average Precision (AP) or Mean Average Precision (mAP) metric. mAP is a commonly used evaluation metric in computer vision for measuring the accuracy of instance segmentation and object detection models. Many of the state-of-the-art object detection algorithms, such as Faster R-CNN[41], Mask R-CNN[42], MobileNet SSD[43], and

YOLO[44], and benchmark challenges such as PASCAL VOC[45], use AP to evaluate their models. Calculation of AP is dependent on the following metrics:

Precision: It is defined as the fraction of true instances among all predicted instances and is calculated using the following formula:

$$\text{Precision} = \frac{TP}{TP + FP}$$

Recall: It is a metric that represents the fraction of retrieved instances among all relevant instances and is calculated as follows:

$$\text{Recall} = \frac{TP}{TP + FN}$$

Intersection over Union (IoU): The intersection over union (IoU) is a metric that measures the overlap between two bounding boxes or masks. It is commonly used to evaluate the accuracy of object detection and instance segmentation models. The IoU value ranges from 0 to 1, with a value of 1 indicating a completely accurate prediction. To calculate the IoU, the overlap between the predicted and ground truth regions is first determined and divided by the total area of both regions. The IoU is a useful metric because it allows for comparing predictions with different shapes and sizes, as it considers the area of both the predicted and ground truth regions (Figure 3B).

Average Precision (AP): The Average Precision (AP) is a metric used to evaluate the performance of object detection and instance segmentation models. It is calculated as the area under the precision-recall curve, which plots the precision (the proportion of true positive detections among all positive detections) against the recall (the proportion of true positive detections among all ground truth objects) of a model. AP ranges from 0 to 1, with a higher value indicating better performance. A higher AP value indicates that the model can achieve both high precision and high recall, making it a useful metric for evaluating the overall performance of a model. AP can be calculated for a specific IoU threshold as follows:

$$\text{AP} = \int_0^1 \text{Precision d(Recall)}$$

Often, AP is used as the average over multiple IoU thresholds, and it is calculated as follows:

$$\text{mAP} = \frac{1}{n}\sum_{k=1}^{k=n} AP_k$$

where,

AP_k = AP at k^{th} IoU threshold

n = Number of IoU thresholds under consideration.

In the following, $AP_{@0.75}$ represents AP at IoU threshold 0.75 and $AP_{@[0.5:0.95]}$ represents the average AP over 10 IoU thresholds (from 0.5 to 0.95 with a step size of 0.05).

Area and Intensity Calculation

After performing object detection and instance segmentation on an image, we can use the predicted contour boundary of each spheroid to calculate its area, circularity, and intensity. To calculate the area of a spheroid, we use Python's OpenCV library to count the number of pixels within the contour boundary. This gives us the total area of the spheroid in pixels. To calculate the intensity of the spheroid, we follow a similar process. First, we create a new image with the same shape and number of pixels as the original, but with a default intensity of zero. This image is then masked with the predicted contour boundary of the spheroid, setting all pixels within the boundary to a value of 255. We then extract the *x* and *y* coordinates of all pixels with a value of 255, which correspond to the pixels within the contour boundary of the spheroid in the original image. Finally, we use OpenCV to calculate the average intensity of these pixels, which gives us the intensity value for the spheroid. This process allows us to accurately measure the area and intensity of each spheroid in an image (Figure 3C).

Data Availability

The source code, example input data, and a detailed tutorial for SpheroScan are available at Github [46]. All supporting data, which includes images used for training, validation, and testing [47], as well as the trained model weights [48], is available at zenodo. Additionally, spheroid images from the external datasets that have been used to evaluate the applicability of SpheroScan, along with the corresponding masked images, are also available at Zenodo [49]. An archival copy of the SpheroScan code is available via the Gigascience database GigaDB [50].

Availability of supporting source code and requirements

Project name: SpheroScan

Project home page: https://github.com/FunctionalUrology/SpheroScan

BioTool ID: spheroscan

SciCrunch ID: SpheroScan (RRID:SCR_023886)

Operating system(s): Linux or Mac

Programming language: Python 3.10.6

Other requirements: Docker, Python, Anaconda, Git

License: GNU GPL

Author contributions statement

K.M., A.H.G, and A.A. conceived the idea for the manuscript. M.B generated all the data. A.A. developed the deep learning pipeline. A.A and M.K developed the code for SpheroScan. K.M., F.C.B, and A.H.G tested the SpheroScan and provided scientific inputs throughout the development phase. F.C.B, R.M.A and A.B.A provided the feedback on biological application of the tool. N.S and M.A provided the mathematical support and did the testing and debugging. All authors contributed to writing, proofreading, and correcting the manuscript.

Funding

We gratefully acknowledge the financial support of the Swiss National Science Foundation (SNF Grant 310030_175773 to FCB and KM, 212298 to FCB and AHG) and the Wings for Life Spinal Cord Research Foundation (WFL-AT-06/19 to KM). AHG and RMA are supported by R01 DK 077195 and R01 DK127673. MK is supported by the Else Kröner-Fresenius-Stiftung (EKFS 2021_EKeA.33). The authors acknowledge the financial support from the Federal Ministry of Education and Research of Germany and by the Sächsische Staatsministerium für Wissenschaft Kultur und Tourismus in the program Center of Excellence for AI-research "Center for Scalable Data Analytics and Artificial Intelligence Dresden/Leipzig" (project identification number: ScaDS.AI).

Conflict of Interest

The authors have declared no competing interests.

Acknowledgments

We are thankful to Ankush Sharma for his guidance and to Niharika Jakhar for testing SpheroScan on various operating systems.

References

(1) Brüningk, S. C.; Rivens, I.; Box, C.; Oelfke, U.; ter Haar, G. 3D Tumour Spheroids for the Prediction of the Effects of Radiation and Hyperthermia Treatments. *Sci Rep* **2020**, *10* (1), 1653. https://doi.org/10.1038/s41598-020-58569-4.

(2) Mehta, G.; Hsiao, A. Y.; Ingram, M.; Luker, G. D.; Takayama, S. Opportunities and Challenges for Use of Tumor Spheroids as Models to Test Drug Delivery and Efficacy. *Journal of Controlled Release* **2012**, *164* (2), 192–204. https://doi.org/10.1016/j.jconrel.2012.04.045.

(3) Carragher, N.; Piccinini, F.; Tesei, A.; Jr, O. J. T.; Bickle, M.; Horvath, P. Concerns, Challenges and Promises of High-Content Analysis of 3D Cellular Models. *Nat Rev Drug Discov* **2018**, *17* (8), 606–606. https://doi.org/10.1038/nrd.2018.99.

(4) Smalley, K. S.; Lioni, M.; Noma, K.; Haass, N. K.; Herlyn, M. In Vitro Three-Dimensional Tumor Microenvironment Models for Anticancer Drug Discovery. *Expert Opinion on Drug Discovery* **2008**, *3* (1), 1–10. https://doi.org/10.1517/17460441.3.1.1.

(5) Spoerri, L.; Gunasingh, G.; Haass, N. K. Fluorescence-Based Quantitative and Spatial Analysis of Tumour Spheroids: A Proposed Tool to Predict Patient-Specific Therapy Response. *Frontiers in Digital Health* **2021**, *3*.

(6) Zhang, Q.; Wang, P.; Fang, X.; Lin, F.; Fang, J.; Xiong, C. Collagen Gel Contraction Assays: From Modelling Wound Healing to Quantifying Cellular Interactions with Three-Dimensional Extracellular Matrices. *European Journal of Cell Biology* **2022**, *101* (3), 151253. https://doi.org/10.1016/j.ejcb.2022.151253.

(7) Moraes, C.; Simon, A. B.; Putnam, A. J.; Takayama, S. Aqueous Two-Phase Printing of Cell-Containing Contractile Collagen Microgels. *Biomaterials* **2013**, *34* (37), 9623–9631. https://doi.org/10.1016/j.biomaterials.2013.08.046.

(8) Yamanishi, C.; Parigoris, E.; Takayama, S. Kinetic Analysis of Label-Free Microscale Collagen Gel Contraction Using Machine Learning-Aided Image Analysis. *Frontiers in Bioengineering and Biotechnology* **2020**, *8*.

(9) Hoque, M. T.; Windus, L. C. E.; Lovitt, C. J.; Avery, V. M. PCaAnalyser: A 2D-Image Analysis Based Module for Effective Determination of Prostate Cancer Progression in 3D Culture. *PLOS ONE* **2013**, *8* (11), e79865. https://doi.org/10.1371/journal.pone.0079865.

(10) Ivanov, D. P.; Parker, T. L.; Walker, D. A.; Alexander, C.; Ashford, M. B.; Gellert, P. R.; Garnett, M. C. Multiplexing Spheroid Volume, Resazurin and Acid Phosphatase Viability Assays for High-Throughput Screening of Tumour Spheroids and Stem Cell Neurospheres. *PLOS ONE* **2014**, *9* (8), e103817. https://doi.org/10.1371/journal.pone.0103817.

(11) Chen, W.; Wong, C.; Vosburgh, E.; Levine, A. J.; Foran, D. J.; Xu, E. Y. High-Throughput Image Analysis of Tumor Spheroids: A User-Friendly Software Application to Measure the Size of Spheroids Automatically and Accurately. *JoVE (Journal of Visualized Experiments)* **2014**, No. 89, e51639. https://doi.org/10.3791/51639.

(12) Piccinini, F. AnaSP: A Software Suite for Automatic Image Analysis of Multicellular Spheroids. *Computer Methods and Programs in Biomedicine* **2015**, *119* (1), 43–52. https://doi.org/10.1016/j.cmpb.2015.02.006.
(13) Monjaret, F.; Fernandes, M.; Duchemin-Pelletier, E.; Argento, A.; Degot, S.; Young, J. Fully Automated One-Step Production of Functional 3D Tumor Spheroids for High-Content Screening. *SLAS Technology* **2016**, *21* (2), 268–280. https://doi.org/10.1177/2211068215607058.
(14) Rueden, C. T.; Schindelin, J.; Hiner, M. C.; DeZonia, B. E.; Walter, A. E.; Arena, E. T.; Eliceiri, K. W. ImageJ2: ImageJ for the next Generation of Scientific Image Data. *BMC Bioinformatics* **2017**, *18* (1), 529. https://doi.org/10.1186/s12859-017-1934-z.
(15) Moriconi, C.; Palmieri, V.; Di Santo, R.; Tornillo, G.; Papi, M.; Pilkington, G.; De Spirito, M.; Gumbleton, M. INSIDIA: A FIJI Macro Delivering High-Throughput and High-Content Spheroid Invasion Analysis. *Biotechnology Journal* **2017**, *12* (10), 1700140. https://doi.org/10.1002/biot.201700140.
(16) Roerdink, J. B. T. M.; Meijster, A. The Watershed Transform: Definitions, Algorithms and Parallelization Strategies. *Fundam. Inf.* **2000**, *41* (1,2), 187–228.
(17) Otsu, N. A Threshold Selection Method from Gray-Level Histograms. *IEEE Transactions on Systems, Man, and Cybernetics* **1979**, *9* (1), 62–66. https://doi.org/10.1109/TSMC.1979.4310076.
(18) Yen, J.-C.; Chang, F.-J.; Chang, S. A New Criterion for Automatic Multilevel Thresholding. *IEEE Transactions on Image Processing* **1995**, *4* (3), 370–378. https://doi.org/10.1109/83.366472.
(19) Duda, R. O.; Hart, P. E. Use of the Hough Transformation to Detect Lines and Curves in Pictures. *Commun. ACM* **1972**, *15* (1), 11–15. https://doi.org/10.1145/361237.361242.
(20) Caselles, V.; Kimmel, R.; Sapiro, G. Geodesic Active Contours. *International Journal of Computer Vision* **1997**, *22* (1), 61–79. https://doi.org/10.1023/A:1007979827043.
(21) Salau, J.; Krieter, J. Instance Segmentation with Mask R-CNN Applied to Loose-Housed Dairy Cows in a Multi-Camera Setting. *Animals* **2020**, *10* (12), 2402. https://doi.org/10.3390/ani10122402.
(22) Hong, Y.; Han, H.-J.; Lee, H.; Lee, D.; Ko, J.; Hong, Z.; Lee, J.-Y.; Seok, J.-H.; Lim, H. S.; Son, W.-C.; Sohn, I. Deep Learning Method for Comet Segmentation and Comet Assay Image Analysis. *Sci Rep* **2020**, *10* (1), 18915. https://doi.org/10.1038/s41598-020-75592-7.
(23) Sun, J.; Tárnok, A.; Su, X. Deep Learning-Based Single-Cell Optical Image Studies. *Cytometry Part A* **2020**, *97* (3), 226–240. https://doi.org/10.1002/cyto.a.23973.
(24) Fudickar, S.; Nustede, E. J.; Dreyer, E.; Bornhorst, J. Mask R-CNN Based C. Elegans Detection with a DIY Microscope. *Biosensors* **2021**, *11* (8), 257. https://doi.org/10.3390/bios11080257.
(25) Beleon, A.; Pignatta, S.; Arienti, C.; Carbonaro, A.; Horvath, P.; Martinelli, G.; Castellani, G.; Tesei, A.; Piccinini, F. CometAnalyser: A User-Friendly, Open-Source Deep-Learning Microscopy Tool for Quantitative Comet Assay Analysis. *Computational and Structural Biotechnology Journal* **2022**, *20*, 4122–4130. https://doi.org/10.1016/j.csbj.2022.07.053.
(26) Abdul, L.; Rajasekar, S.; Lin, D. S. Y.; Raja, S. V.; Sotra, A.; Feng, Y.; Liu, A.; Zhang, B. Deep-LUMEN Assay – Human Lung Epithelial Spheroid Classification

from Brightfield Images Using Deep Learning. *Lab Chip* **2020**, *20* (24), 4623–4631. https://doi.org/10.1039/D0LC01010C.
(27) Lacalle, D.; Castro-Abril, H. A.; Randelovic, T.; Domínguez, C.; Heras, J.; Mata, E.; Mata, G.; Méndez, Y.; Pascual, V.; Ochoa, I. SpheroidJ: An Open-Source Set of Tools for Spheroid Segmentation. *Computer Methods and Programs in Biomedicine* **2021**, *200*, 105837. https://doi.org/10.1016/j.cmpb.2020.105837.
(28) Grexa, I.; Diosdi, A.; Harmati, M.; Kriston, A.; Moshkov, N.; Buzas, K.; Pietiäinen, V.; Koos, K.; Horvath, P. SpheroidPicker for Automated 3D Cell Culture Manipulation Using Deep Learning. *Sci Rep* **2021**, *11* (1), 14813. https://doi.org/10.1038/s41598-021-94217-1.
(29) Chen, Z.; Ma, N.; Sun, X.; Li, Q.; Zeng, Y.; Chen, F.; Sun, S.; Xu, J.; Zhang, J.; Ye, H.; Ge, J.; Zhang, Z.; Cui, X.; Leong, K.; Chen, Y.; Gu, Z. Automated Evaluation of Tumor Spheroid Behavior in 3D Culture Using Deep Learning-Based Recognition. *Biomaterials* **2021**, *272*, 120770. https://doi.org/10.1016/j.biomaterials.2021.120770.
(30) Trossbach, M.; Åkerlund, E.; Langer, K.; Seashore-Ludlow, B.; Joensson, H. N. High-Throughput Cell Spheroid Production and Assembly Analysis by Microfluidics and Deep Learning. *SLAS Technology* **2023**. https://doi.org/10.1016/j.slast.2023.03.003.
(31) Piccinini, F.; Peirsman, A.; Stellato, M.; Pyun, J.-C.; Tumedei, M. M.; Tazzari, M.; Wever, O. D.; Tesei, A.; Martinelli, G.; Castellani, G. Deep Learning-Based Tool for Morphotypic Analysis of 3d Multicellular Spheroids. *J. Mech. Med. Biol.* **2023**, 2340034. https://doi.org/10.1142/S0219519423400341.
(32) Peirsman, A.; Blondeel, E.; Ahmed, T.; Anckaert, J.; Audenaert, D.; Boterberg, T.; Buzas, K.; Carragher, N.; Castellani, G.; Castro, F.; Dangles-Marie, V.; Dawson, J.; De Tullio, P.; De Vlieghere, E.; Dedeyne, S.; Depypere, H.; Diosdi, A.; Dmitriev, R. I.; Dolznig, H.; Fischer, S.; Gespach, C.; Goossens, V.; Heino, J.; Hendrix, A.; Horvath, P.; Kunz-Schughart, L. A.; Maes, S.; Mangodt, C.; Mestdagh, P.; Michlíková, S.; Oliveira, M. J.; Pampaloni, F.; Piccinini, F.; Pinheiro, C.; Rahn, J.; Robbins, S. M.; Siljamäki, E.; Steigemann, P.; Sys, G.; Takayama, S.; Tesei, A.; Tulkens, J.; Van Waeyenberge, M.; Vandesompele, J.; Wagemans, G.; Weindorfer, C.; Yigit, N.; Zablowsky, N.; Zanoni, M.; Blondeel, P.; De Wever, O. MISpheroID: A Knowledgebase and Transparency Tool for Minimum Information in Spheroid Identity. *Nat Methods* **2021**, *18* (11), 1294–1303. https://doi.org/10.1038/s41592-021-01291-4.
(33) Diosdi, A.; Hirling, D.; Kovacs, M.; Toth, T.; Harmati, M.; Koos, K.; Buzas, K.; Piccinini, F.; Horvath, P. Cell Lines and Clearing Approaches: A Single-Cell Level 3D Light-Sheet Fluorescence Microscopy Dataset of Multicellular Spheroids. *Data in Brief* **2021**, *36*, 107090. https://doi.org/10.1016/j.dib.2021.107090.
(34) Nürnberg, E.; Vitacolonna, M.; Klicks, J.; von Molitor, E.; Cesetti, T.; Keller, F.; Bruch, R.; Ertongur-Fauth, T.; Riedel, K.; Scholz, P.; Lau, T.; Schneider, R.; Meier, J.; Hafner, M.; Rudolf, R. Routine Optical Clearing of 3D-Cell Cultures: Simplicity Forward. *Frontiers in Molecular Biosciences* **2020**, *7*.
(35) Hossain, S. Visualization of Bioinformatics Data with Dash Bio. *Proceedings of the 18th Python in Science Conference* **2019**, 126–133. https://doi.org/10.25080/Majora-7ddc1dd1-012.
(36) The pandas development team. Pandas-Dev/Pandas: Pandas, 2020. https://doi.org/10.5281/zenodo.3509134.

(37) McKinney, W. Data Structures for Statistical Computing in Python. *Proceedings of the 9th Python in Science Conference* **2010**, 56–61. https://doi.org/10.25080/Majora-92bf1922-00a.
(38) Dutta, A.; Zisserman, A. The VIA Annotation Software for Images, Audio and Video. In *Proceedings of the 27th ACM International Conference on Multimedia*; MM ’19; Association for Computing Machinery: New York, NY, USA, 2019; pp 2276–2279. https://doi.org/10.1145/3343031.3350535.
(39) van Rossum, G. Python Reference Manual. **1995**.
(40) Wu, Y.; Kirillov, A.; Massa, F.; Lo, W.-Y.; Girshick, R. Detectron2, 2019. https://github.com/facebookresearch/detectron2.
(41) Ren, S.; He, K.; Girshick, R.; Sun, J. Faster R-CNN: Towards Real-Time Object Detection with Region Proposal Networks. *IEEE Transactions on Pattern Analysis and Machine Intelligence* **2017**, *39* (6), 1137–1149. https://doi.org/10.1109/TPAMI.2016.2577031.
(42) He, K.; Gkioxari, G.; Dollár, P.; Girshick, R. Mask R-CNN. In *2017 IEEE International Conference on Computer Vision (ICCV)*; 2017; pp 2980–2988. https://doi.org/10.1109/ICCV.2017.322.
(43) Howard, A. G.; Zhu, M.; Chen, B.; Kalenichenko, D.; Wang, W.; Weyand, T.; Andreetto, M.; Adam, H. MobileNets: Efficient Convolutional Neural Networks for Mobile Vision Applications. arXiv April 16, 2017. https://doi.org/10.48550/arXiv.1704.04861.
(44) Redmon, J.; Divvala, S.; Girshick, R.; Farhadi, A. You Only Look Once: Unified, Real-Time Object Detection. In *2016 IEEE Conference on Computer Vision and Pattern Recognition (CVPR)*; 2016; pp 779–788. https://doi.org/10.1109/CVPR.2016.91.
(45) Everingham, M.; Van Gool, L.; Williams, C. K. I.; Winn, J.; Zisserman, A. The Pascal Visual Object Classes (VOC) Challenge. *Int J Comput Vis* **2010**, *88* (2), 303–338. https://doi.org/10.1007/s11263-009-0275-4.
(46) SpheroScan software repository. Github. 2023. https://github.com/FunctionalUrology/SpheroScan .
(47) Akshay, A.; Katoch, M.; Abedi, M.; Besic, M.; Shekarchizadeh, N.; Burkhard, F. C.; Bigger-Allen, A.; Adam, R. M.; Monastyrskaya, K.; Gheinani, A. H. Supporting Data for “SpheroScan: A User-Friendly Deep Learning Tool for Spheroid Image Analysis,” 2023. https://doi.org/10.5281/zenodo.7555467.
(48) Akshay, A.; Katoch, M.; Abedi, M.; Besic, M.; Shekarchizadeh, N.; Burkhard, F. C.; Bigger-Allen, A.; Adam, R. M.; Monastyrskaya, K.; Gheinani, A. H. Trained Model Weights for “SpheroScan: A User-Friendly Deep Learning Tool for Spheroid Image Analysis.” **2023**. https://doi.org/10.5281/zenodo.7552508.
(49) Akshay, A.; Katoch, M.; Abedi, M.; Besic, M.; Shekarchizadeh, N.; Burkhard, F. C.; Bigger-Allen, A.; Adam, R. M.; Monastyrskaya, K.; Gheinani, A. H. External Test Datasets for “SpheroScan: A User-Friendly Deep Learning Tool for Spheroid Image Analysis,” 2023. https://doi.org/10.5281/zenodo.8211845.
(50) Akshay A, Katoch M, Abedi M, Shekarchizadeh N, Besic M, Burkhard FC et al. Supporting data for "SpheroScan: A User-Friendly Deep Learning Tool for Spheroid Image Analysis" GigaScience Database. 2023 http://dx.doi.org/10.5524/102444

Click here to access/download
Supplementary Material
supplementary_AHG.docx

Click here to access/download
Supplementary Material
figGallery_suppl_20230807.pdf
