# Supplementary material for: SpheroScan: a user-friendly deep learning tool for spheroid image analysis
Source: Gigascience. 2023 Oct 27;12:giad082. doi: 10.1093/gigascience/giad082 (PMC10603766; doi:10.1093/gigascience/giad082)
Supplement: giad082_Supplemental_Files [file giad082_supplemental_files.zip › figGallery_suppl_20230807.pdf]

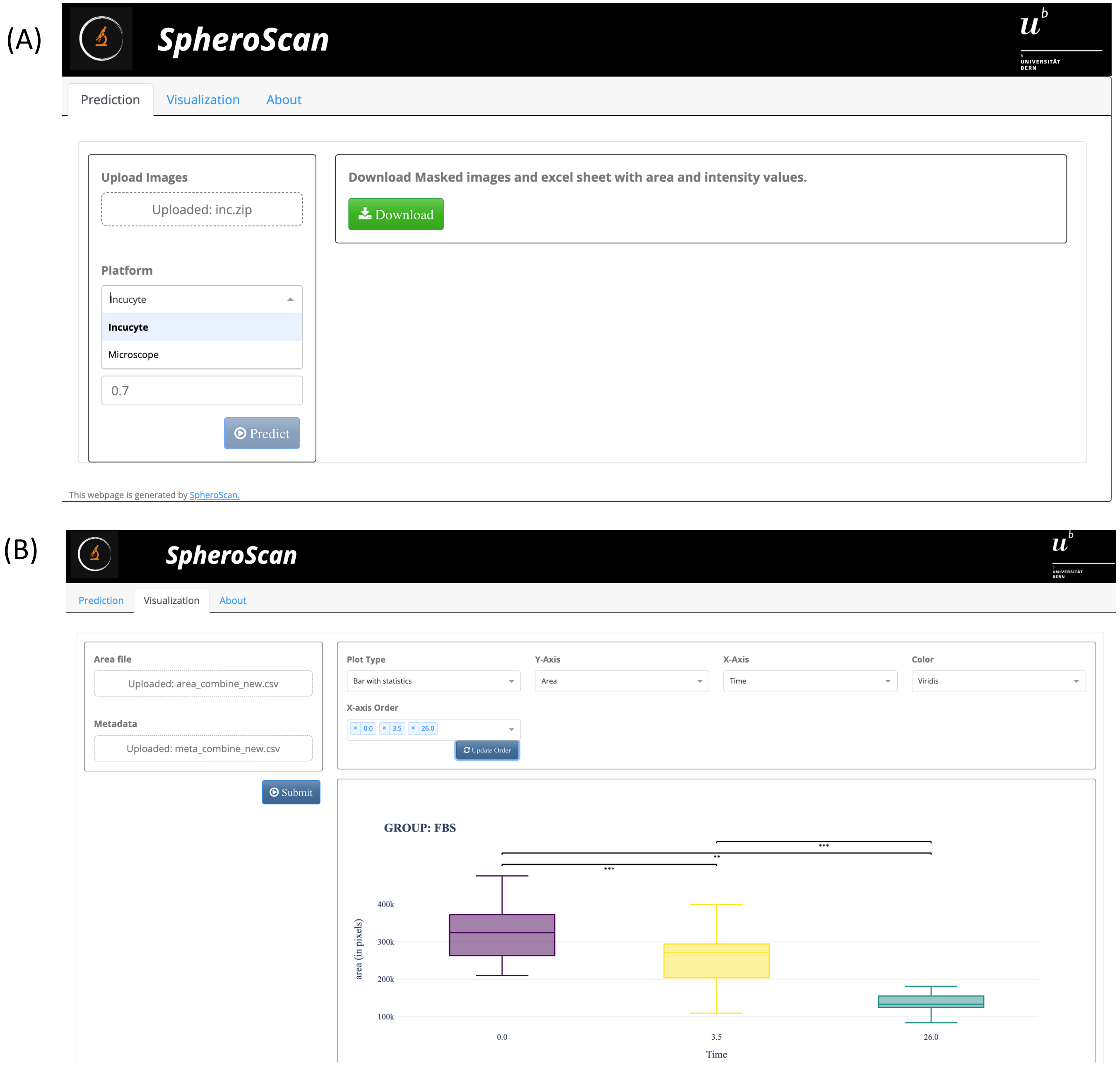


**Figure S1. SpheroScan Graphical User Interface. (A) Prediction Module.** The Prediction Module applies trained DL models to identify and mask spheroid images. It requires a zipped folder of images, platform type, and prediction threshold as input and generates masked images and a CSV file containing the area and intensity data of the identified spheroids as output. **(B) Visualization Module.** The Visualization Module creates plots and performs statistical analysis using the output file from the Prediction Module and a metadata file that contains information about the study design. It offers various types of plots and allows users to customize the plot options, such as plot type and color palette. Users can export plots in high-resolution PNG format.

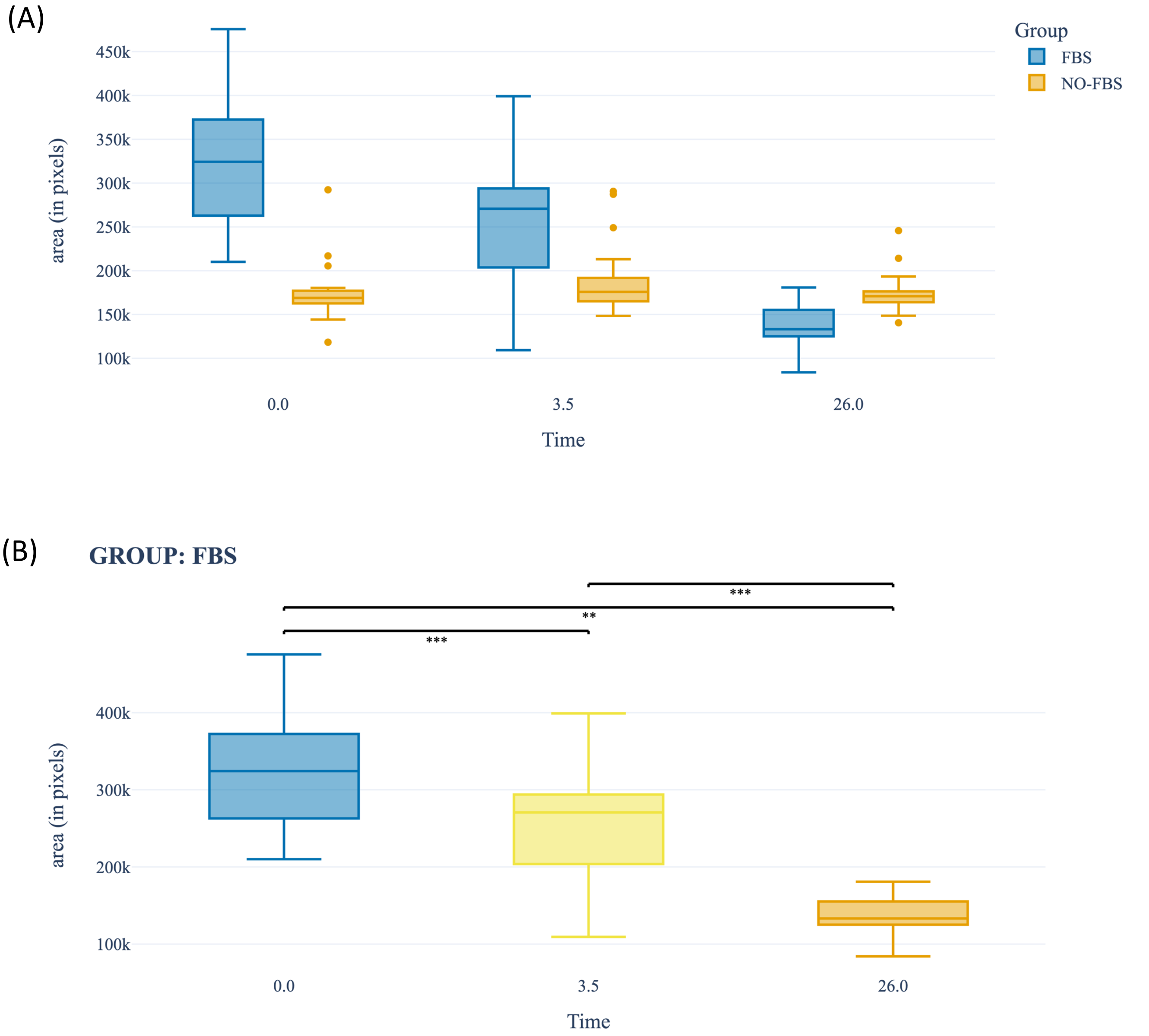


**Figure S2. SpheroScan Plot Gallery. (A)** Bar Plot **(B)** Bar Plot with significance level. A bar plot with significance level is a visual representation of data where the level of significance is indicated by stars. Three stars (***) indicate a p-value of less than 0.001, while "ns" represents a p-value of 0.05 or greater. The less stars, the lower the significance level.

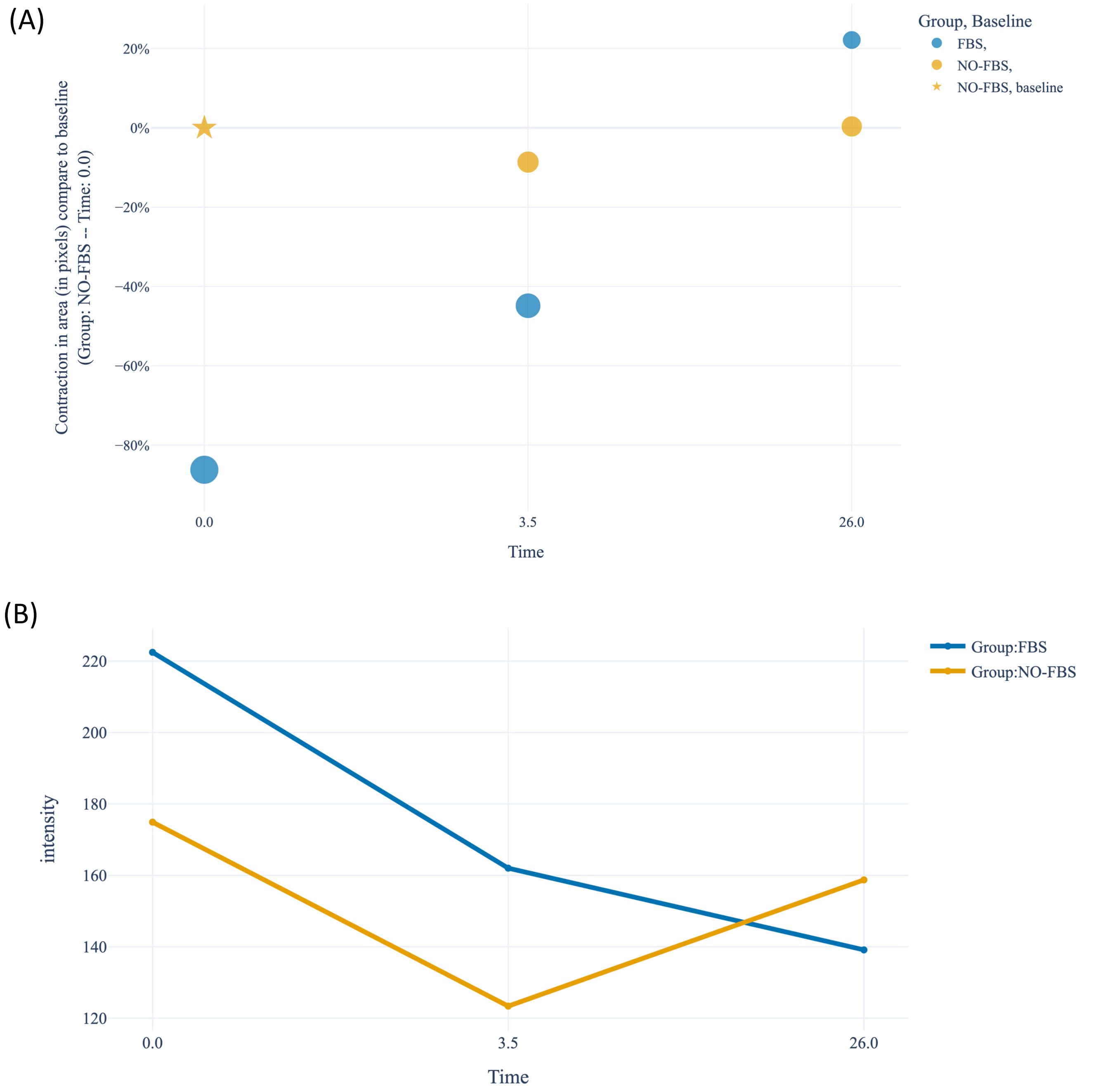


**Figure S3. SpheroScan Plot Gallery. (A)** Bubble Plot. A bubble plot is a type of scatter plot where the size of the bubbles represents the mean spheroid area for a certain group. The Y-axis displays the relative area or contraction of the spheroid, calculated with respect to a baseline group. **(B)** Line plot

(A)

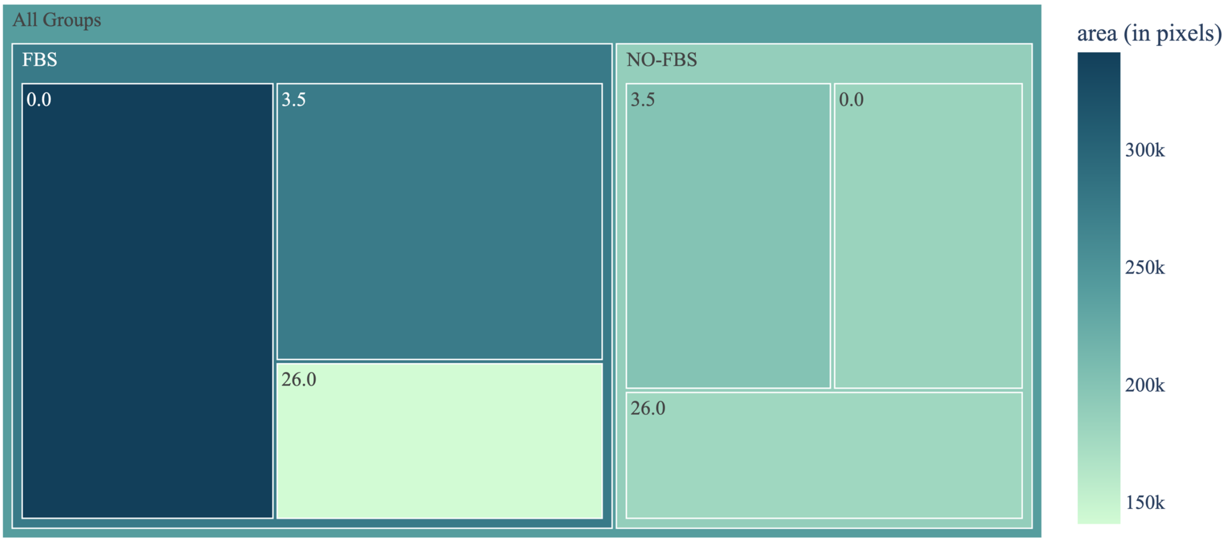


(B)

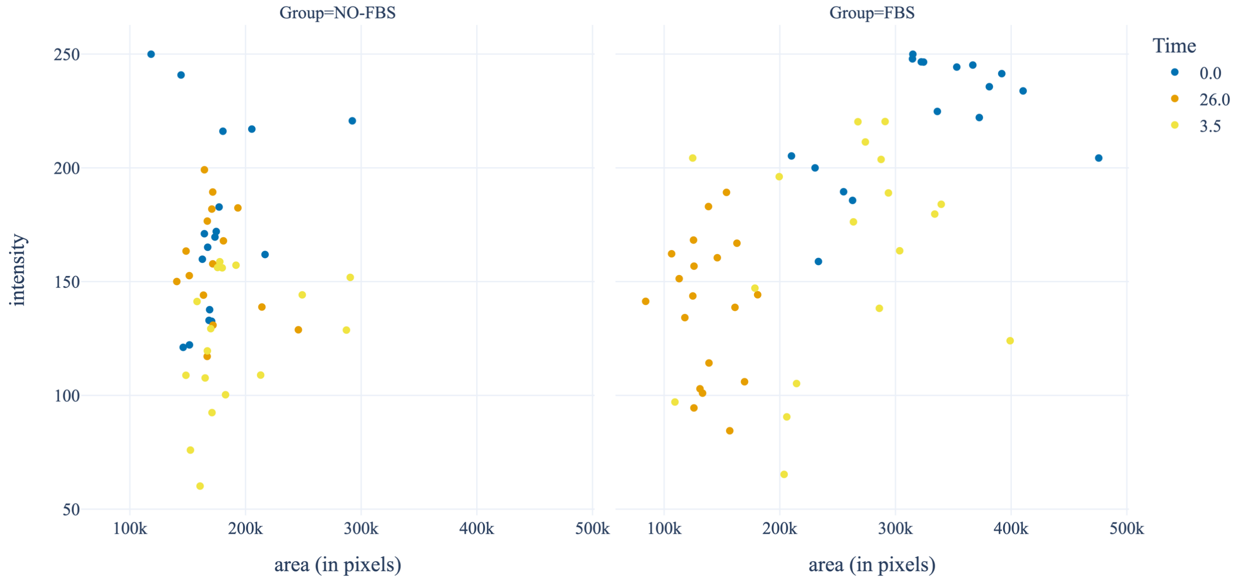


**Figure S4. SpheroScan Plot Gallery. (A)** Treemap. A treemap is a method of displaying hierarchical data in which nested rectangles are used to represent different groups. The outer rectangles represent the top-level groups, while the inner rectangles represent sub-groups. The size and color of each rectangle in the treemap indicate the mean spheroid areas or intensity of the corresponding group. **(B)** Scatter plot

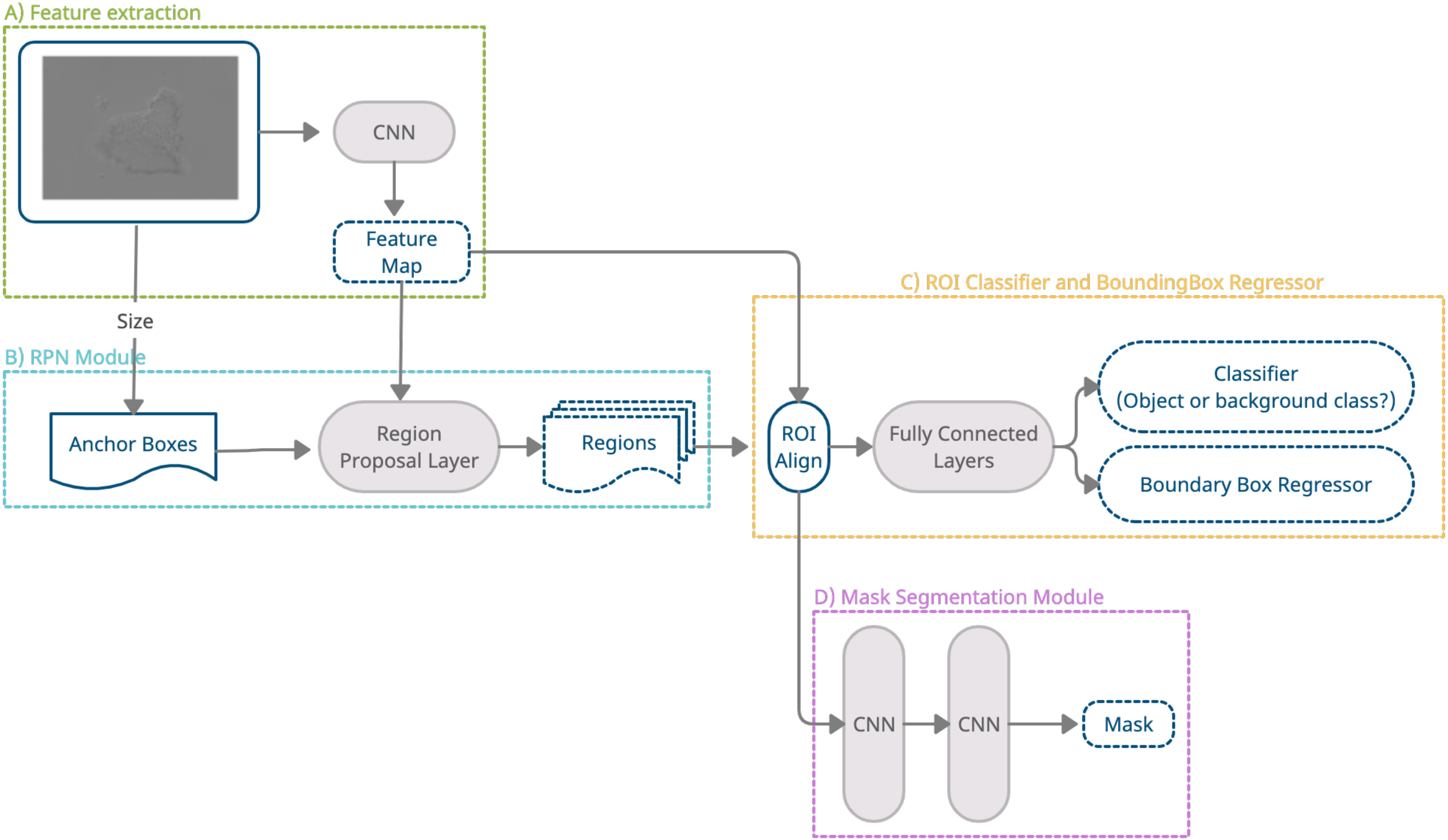


**Figure S5. Mask R-CNN Architecture.** The Mask R-CNN model consists of four main modules: feature extraction, Region Proposal Network (RPN), Region of Interest (ROI) classifier and bounding box regressor, and mask segmentation. The feature extraction module takes images as input and produces feature maps. The RPN module then runs on the feature maps and uses a sliding window to identify bounding boxes with a high likelihood of containing objects (ROIs). For each ROI, the ROI classifier and bounding box regressor module is used to determine the class label of the object. For semantic segmentation, the Mask R-CNN model uses a Fully Convolutional Network (FCN) in the mask segmentation module to predict a mask for each ROI identified in the object detection phase.

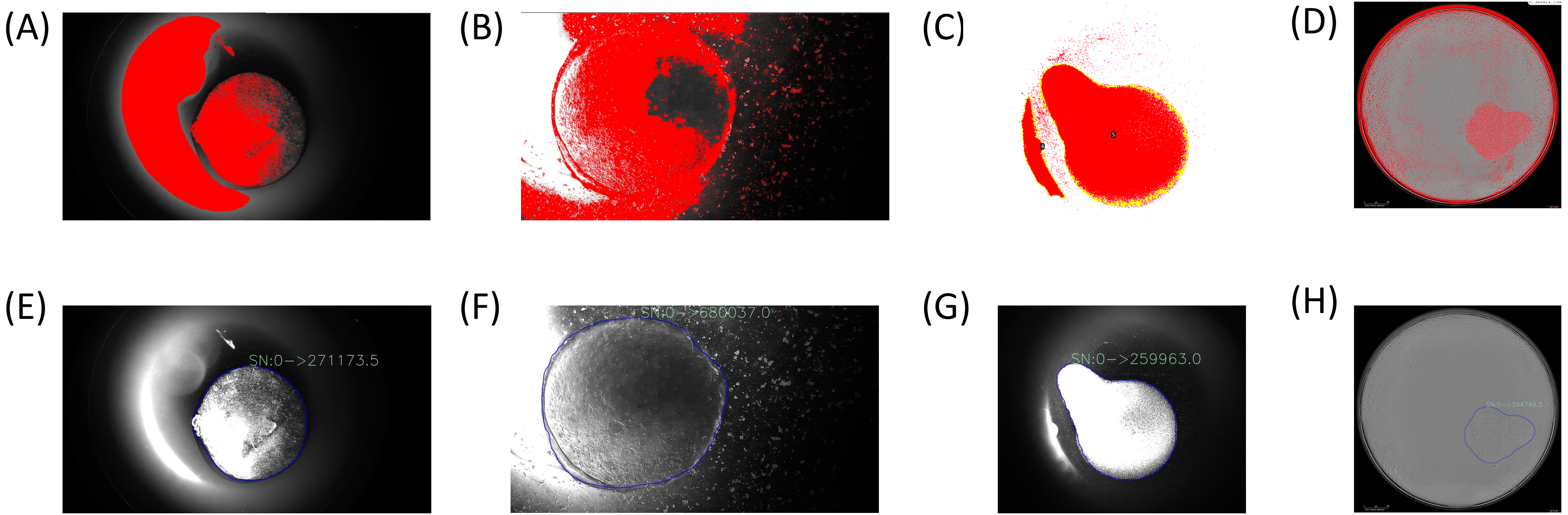


**Figure S6: A comparison between masking methods: Thresholding approach versus SpheroScan. (A) - (D)** Images masked using the thresholding approach in ImageJ. However, this method proves ineffective in accurately masking the spheroid due to significant contrast variations within the image. **(E) - (F)** Corresponding images masked using SpheroScan, demonstrating more accurate results.

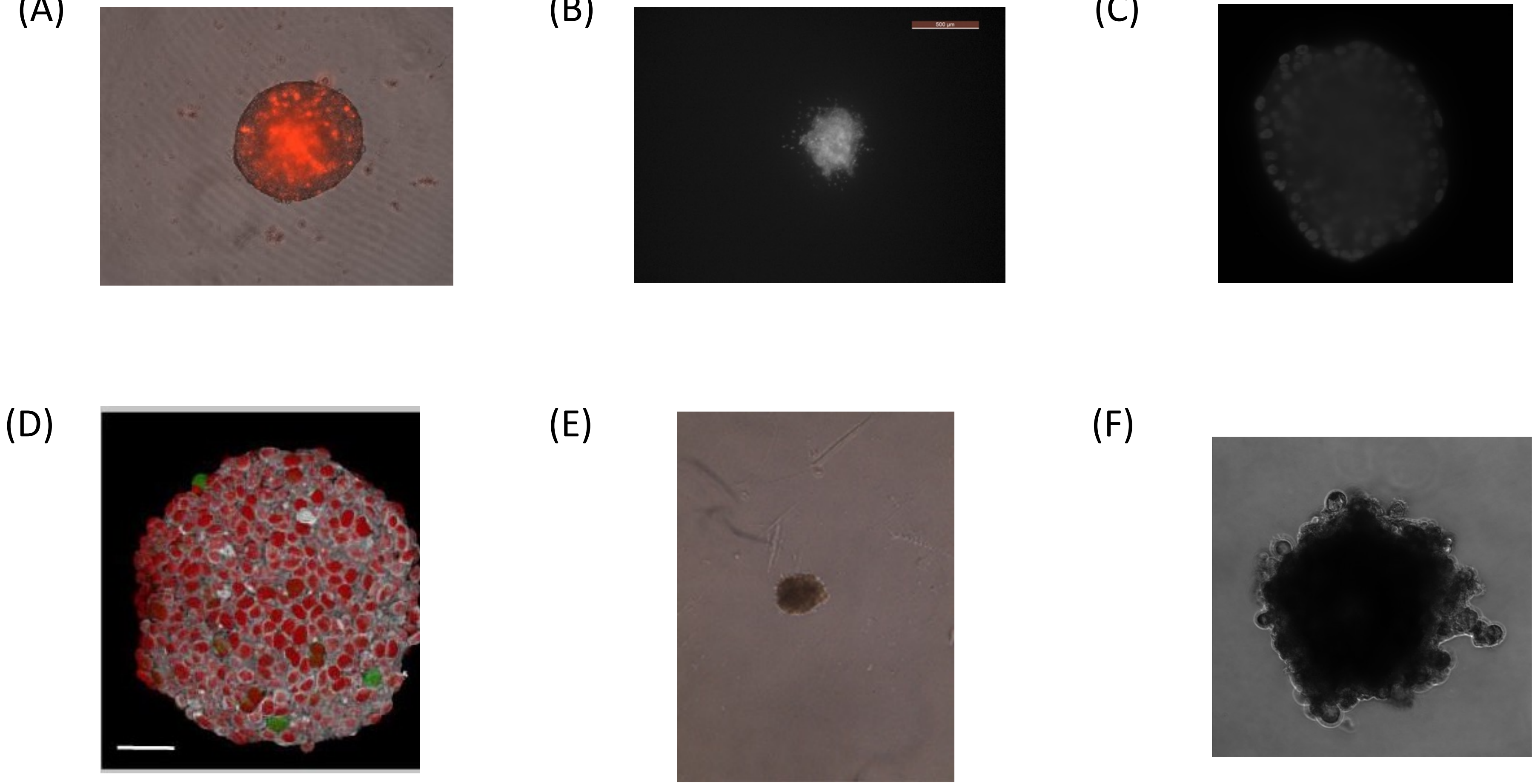


**Figure S7. Sample of spheroid images from External Datasets. (A) - (C)** Fluorescence microscopy images. **(D)** Fluorescence (multichannel) microscopy image. **(E) - (F)** Brightfield microscopy images.

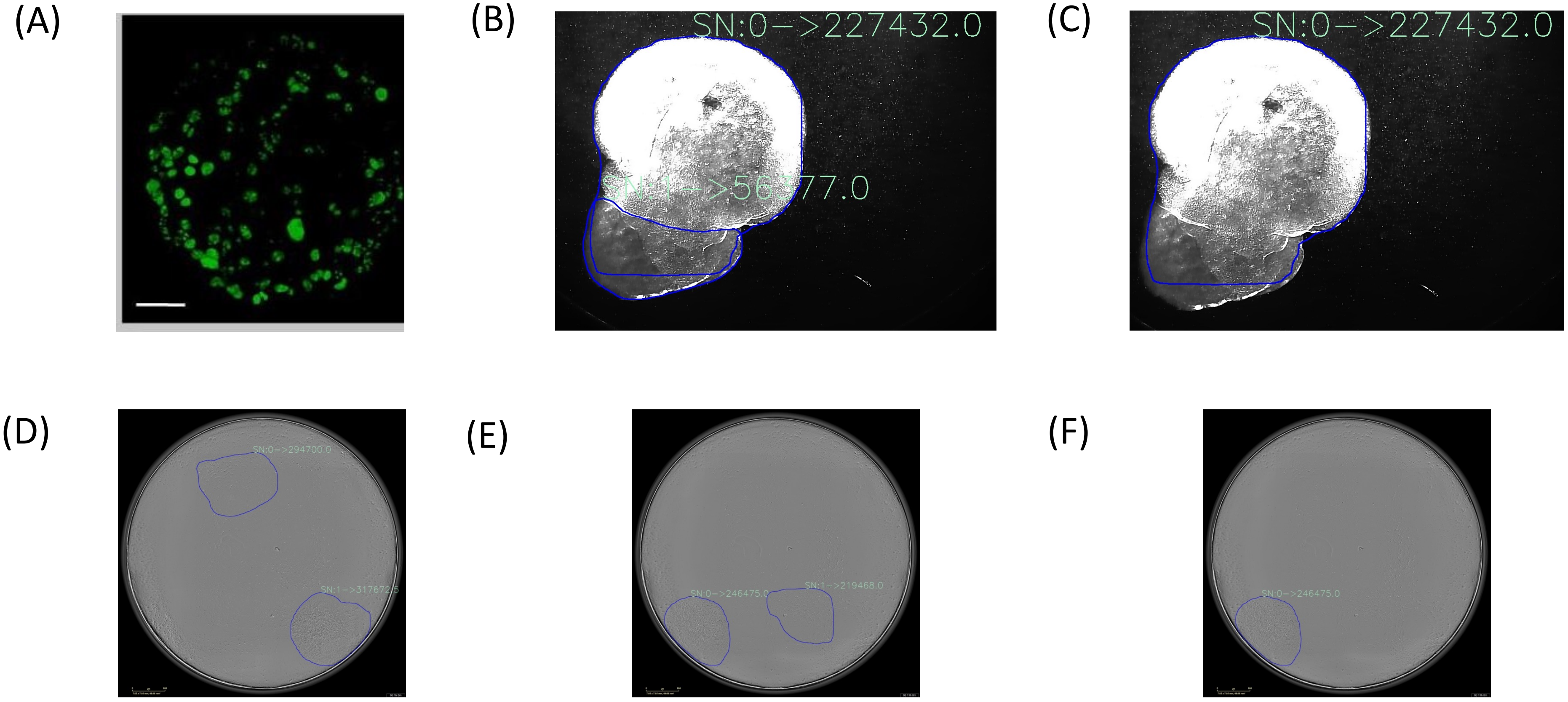


**Figure S8. Challenging scenarios encountered by SpheroScan.** Image **(A)** contains a spheroid formed with a limited number of labelled cells exhibiting hollow, spheroid-like structure, which was not identified by SpheroScan in this image. Images **(B), (D), and (E)** represent spheroid images with debris and irregular shapes, where SpheroScan mistakenly identified debris as spheroids at a prediction threshold of 0.8. To address this issue, the threshold was adjusted to 0.95 for image **(B)** and 0.9 for image **(E)**, leading to correct masking, as shown in Figures **(C)** and **(F)**, respectively. However, even after increasing the threshold, SpheroScan still failed to correctly mask the spheroid in image **(D)**.
