# Supplementary material for: SpheroScan: a user-friendly deep learning tool for spheroid image analysis
Source: Gigascience. 2023 Oct 27;12:giad082. doi: 10.1093/gigascience/giad082 (PMC10603766; doi:10.1093/gigascience/giad082)
Supplement: giad082_Supplemental_Files [file giad082_supplemental_files.zip › supplementary_AHG.docx]

| ***Software*** | ***Web-Server*** | ***GUI*** | ***Interactive Visualization*** | ***Type*** | ***Availaiblity*** |
| --- | --- | --- | --- | --- | --- |
| **SpheroScan** | 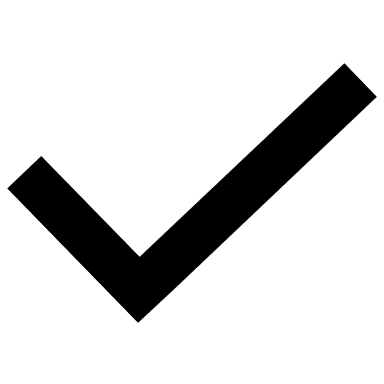 | 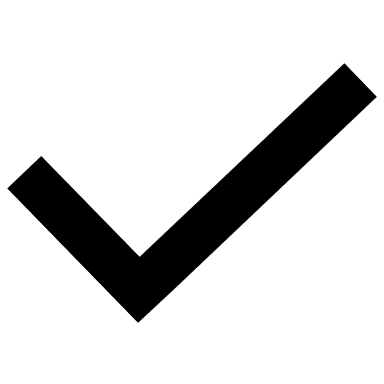 | 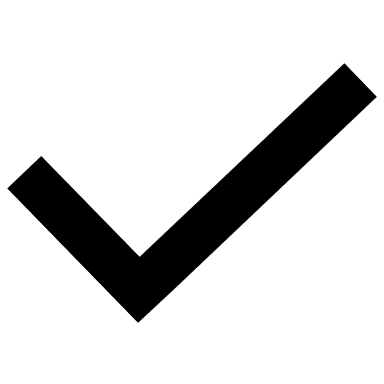 | Standalone | Open source |
| SpheroidPicker |  |  |  | * | On request |
| SpheroidJ |  | 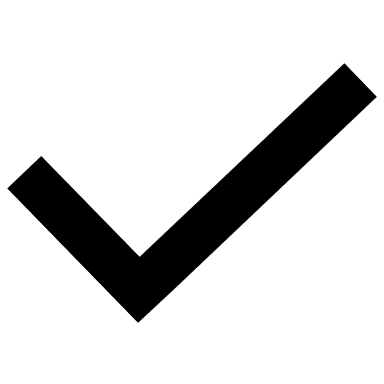 |  | Standalone | Open source |
| AnaSP |  | 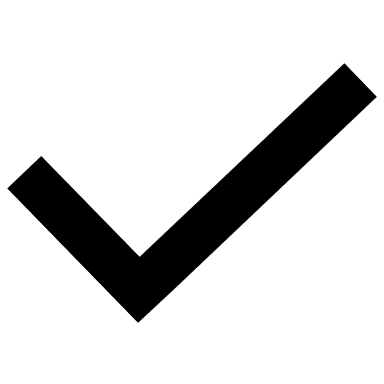 |  | Standalone | Open source |
| SMART |  |  |  | Scripts | Open source |

Table S1: Comparison of features between SpheroScan and other similar deep learning based tools for automatic spheroid detection.* = No information provided in the manuscript. GUI = Graphical User Interface.

| ***Data*** | ***Type*** | ***Microscope*** | ***No of Images*** | ***No of Correctly Masked Images*** |
| --- | --- | --- | --- | --- |
| Peirsman, Arne et al. | Fluorescence | Zeiss | 66 | 66 |
| Diosdi, Akos et al. | Fluorescence | Leica | 42 | 41 |
| Lacalle, David et al. | Fluorescence | Leica | 19 | 19 |
| Nürnberg, Elina et al. | Fluorescence(multi-channel) | Leica | 48 | 40 |
| Ivanov, Delyan P et al. | Brightfield | Olympus | 120 | 120 |
| Lacalle, David et al. | Brightfield | Nikon | 105 | 105 |

Table S2: List of external datasets used to evaluate the performance of SpheroScan on an unseen dataset obtained from various experiments, studies, and conditions.
