# Supplementary material for: SpheroScan: a user-friendly deep learning tool for spheroid image analysis
Source: Gigascience. 2023 Oct 27;12:giad082. doi: 10.1093/gigascience/giad082 (PMC10603766; doi:10.1093/gigascience/giad082)
Supplement: giad082_Response_to_Reviewer_Comments_Original_Submission [file giad082_response_to_reviewer_comments_original_submission.pdf]

Author's Response To Reviewer Comments

Dear Editors and Dear reviewers,

We would like to express our gratitude for the thoughtful review and constructive feedback provided by the reviewers for our
(Manuscript ID: GIGA-D-23-00131). We sincerely appreciate the time and effort invested in evaluating our work, and we are
In light of the reviewers' valuable suggestions, we have carefully considered their comments and have prepared a detailed re

###
Reply to Editor
###
Editor reports:
In particular, the reviewers would like to see additional comparisons and validation (e.g. with external data). Reviewer #2 als
software application in the bio.tools and SciCrunch.org databases to receive RRID (Research Resource Identification Initiative

Answer: Thank you for your valuable feedback. In compliance with your request, we have included these unique identifiers in
supporting source code and requirements.
https://bio.tools/spheroscan
BioTool ID: spheroscan
SciCrunch ID: SpheroScan (RRID:SCR_023886)

###
Reply to Reviewer 1
###

Reviewer reports:
Reviewer #1: The authors present a "Technical Note" about an open-source web tool called SpheroScan. As input users coulc
Prediction Module: creates a file with area and intensity of detected spheroids (CSV), (2) Visualization Module: plots of the co
microscope images with 336 (70 %) and 265 for training, 144 (30 %) and 117 for validation, and 50 images for testing, resp
tested in the range of 0.5 to 0.95 against manual annotation (VGG Annotator). As evaluation measure they used Intersection
calculates values of Average Precision (AP) for masking: 0.937 and 0.972 (Test), 0.927 and 0.97 (Validation) as well as AP fo
proofed with different sized datasets (1 s / image) for masking on a 16 core CPU, 64 GB RAM machine. The tool is available c
General evaluation:
The concept of the tool serves some important needs of 3D cell culture-based assays: automated, standardized, high-through
open how high the impact, the reproducibility, and the chances of potential application by other researchers will be. This is du
well as the (potential) restriction of input data (i.e. brightfield only, not validated with external data) and the limited options
possibility to access a web interface, which is easy to use and will ideally be equipped with additional functionalities in the fut

Answer---------> We thank the reviewer for their insightful comments and valuable feedback on our manuscript. Your comme
your time and efforts in helping to improve the quality of our work.
Regarding "the limitations in accessibility, non-permanent or non-functional web too"l:
We have purchased the infrastructure and incorporated our tool into the web site that can be accessible and tested to be func
Regarding "the (potential) restriction of input data (i.e. brightfield only, not validated with external data) and the limited optio
In response to this constructive comment, the tool SpheroScan was tested for its usability in analyzing spheroid images from
conditions. Added to line 233-244

Comment 1 (minor):
The presented tool uses the Mask R-CNN deep-learning model in their image processing pipeline. Several tools, which perforr
implemented in several commercial imaging devices and allow for segmentation of cell containing image areas, e.g. to determ
also applicable for 2D images of 3D spheroids. The concept of automated image segmentation is thus not novel and only mee
The state-of-the-art and preliminary work are not sufficiently referenced. Several similar and alternative (open-source) tools
2023; Trossbach et al., 2023), to give only a few examples.

Answer---------> Thank you for your valuable feedback. We appreciate your insightful comments regarding the need to provi
have have made significant improvements to the Introduction section to address this concern. Specifically, we have extended
Additionally, we have created Table S1, which provides a comparative summary of similar existing tools and their key feature

----
Comment 2 (major):
The authors claim to present an user-friendly open-source web tool. The python project is available on Github, and on a dem
(https://urldefense.com/v3/__https://spheroscan.onrender.com/__;!!NZvER7FxgEiBAiR_!tgxP4EUU1Jpepc_8xvUk9fyvwMViX
the web interface can be accessed. Unfortunately the mentioned web tool is not functional, i.e. it is stated on the website: "T
prediction functionality, please run SpheroScan on your local machine.". This is significantly limiting the applicability of the pr
demo server should either present a functional user interface (recommended), or the statement should be removed from the

Answer---------> Thank you for your feedback and bringing the issue regarding the functionality of our web tool to our attent
to inform you that the prediction module is now fully active on the server, and the web tool is functional as intended.
Users can access and utilize the web interface without the need to run SpheroScan on their local machines. The web server c
github.

Comment 3 (major):
The presented algorithm was trained exclusively on internal data of brightfield images from "Incucyte and microscope platforr
images.
It remains unclear how the algorithm will perform on external data of prospective users. Given the fact that two distinct mode
robustness of the models in this regard. This is clearly a general problem of image processing algorithms, but one that will st
web tool interface is not functional at this point, the authors will also not be able to evaluate or improve on this after publicat
be performed, to further elaborate on this.

Answer---------> Thank you for providing us with your valuable feedback regarding testing the applicability of SpheroScan or
your input.
To assess the applicability of SpheroScan to external data, we utilized six distinct datasets, including four fluorescence micros
previous studies. These datasets are from various imaging platforms, cell types, growth mediums, and lighting conditions. W
demonstrated that SpheroScan successfully identified spheroids in all images. This validation shows that SpheroScan is adap
techniques. Nevertheless, the Nürnberg, Elina et al. dataset posed a challenge for SpheroScan as it struggled to identify sphe
anti-KI67, resulting in the formation of hollow spheroid-like structures.
We have included the corresponding text in the updated manuscript as well. Please refer to line 233-244. Kindly refer to line

----
Comment 4 (major):
Many assays nowadays use fluorescent labels, for example to calculate cell ratios within 3D arrangements, e.g. for cell viabili
thereof) is or will be able to process multi-channel microscope images of spheroids.
This is a significant limitation of the presented work and should at least be mentioned in the corresponding section, respectiv
algorithm performance and derive potentially necessary adaptations in future versions.

Answer---------> Thank you for providing us with your feedback regarding testing the applicability of SpheroScan to fluoresc
To assess the applicability of SpheroScan fluorescent (multichannel) dataset, we utilized four fluorescence microscopy datase
spheroids in the images from these datasets, and the results demonstrated that SpheroScan successfully identified spheroids
images generated by external users with different imaging techniques. Nevertheless, the Nürnberg, Elina et al. dataset posec
arose from a limited number of cells stained with anti-KI67, resulting in the formation of hollow spheroid-like structures.
We have included the corresponding text in the updated manuscript as well. Please refer to line 233-244. Kindly refer to line
We have also extended the limitation section to address the challenges posed by Nürnberg, Elina et al. dataset. Please refer t

----
Comment 5 (minor):
The output of the tool is a list of detected spheroids with corresponding area (2D) and bright field average intensity within th
case to investigate collagen gel contraction assays. Several other parameters of interest could easily be derived from the me
estimation. This should again be mentioned in the "limitations and considerations" section.

Answer---------> We greatly appreciate your valuable feedback. In response to your comment, we have now included the rou
insights into the spheroid morphology, enhancing the applicability of our tool to a broader range of assays and experimental s
Moreover, we have extended the "limitations and considerations" section in the manuscript to provide a more detailed explan
287 – 293. As we continue to improve the tool, we will consider incorporating these additional parameters in future updates t

###
Reply to Reviewer 2
###

Reviewer #2: This study represents a significant contribution to the field of screening and analysis of three-dimensional cell c
from a large number of spheroids or other types of three-dimensional tissue models is substantial. The authors of this manus
extract the projected area and intensity of individual cellular spheroids imaged with bright-field microscopy. The tool is compa
specimens, typically found in high-density multiwell plates.An admirable aspect of this work is the authors' decision to make
validate the code, promoting transparency and collaboration in the scientific community. However, several improvements sho
One important aspect that the authors should address in the manuscript is the suitability, rationale, and extent of using a ne
segmentation of single bright-field images of spheroids. While neural networks are anticipated to play an increasingly importa
Although there may be segmentation tasks that are challenging to accomplish with traditional approaches, where neural netw
conventional strategies. For example, in our research group, we were able to reliably segment densely populated bright-field
plugin MorphoLibJ (see references:
https://urldefense.com/v3/__https://doi.org/10.1093/bioinformatics/btw413__;!!NZvER7FxgEiBAiR_!tgxP4EUU1Jpepc_8xvU
and
https://urldefense.com/v3/__https://doi.org/10.1186/s12915-021-00958-w__;!!NZvER7FxgEiBAiR_!tgxP4EUU1Jpepc_8xvUk
). Therefore, it would be informative and valuable for readers if the authors compared the results obtained from the neural ne
Watershed) on the same dataset, as demonstrated in a similar study (reference: https://urldefense.com/v3/__https://doi.org
1__;!!NZvER7FxgEiBAiR_!tgxP4EUU1Jpepc_8xvUk9fyvwMViXXppwOWtTyQvxKszSAerS27mDkOkYQRG0KyEEsz6AiI9aRAU12N

Answer---------> Thank you for careful review of our manuscript. Your comments were very helpful, and we have taken them
quality of our work.
We have have made significant improvements to the Introduction section to address this concern. Specifically, we have exter
Furthermore, we have included a figure with example images to demonstrate potential scenarios where thresholding algorith

----

Furthermore, to address the limitations of the model, the authors should provide specific examples (preferably in the supplem
applying the neural network to the data. For instance, it would be beneficial to explore scenarios where spheroids are surrou
situations are common and it is important to provide insights into potential challenges that may arise when the images of the

Answer---------> Thank you for your valuable feedback. We have included a supplementary figure (figure S8) that presents s
limitation section to address this concern. Please refer to line 295 – 305.
