# Supplementary material for: SpheroScan: a user-friendly deep learning tool for spheroid image analysis
Source: Gigascience. 2023 Oct 27;12:giad082. doi: 10.1093/gigascience/giad082 (PMC10603766; doi:10.1093/gigascience/giad082)
Supplement: giad082_Reviewer_1_Report_Original_Submission — Kevin TrÃ¶ndle -- 6/16/2023 Reviewed [file giad082_reviewer_1_report_original_submission.pdf]

Reviewer Report

Title: SpheroScan: A User-Friendly Deep Learning Tool for Spheroid Image Analysis

Version: Original Submission **Date:** 6/16/2023

Reviewer name: Kevin TrÃ¶ndle

Reviewer Comments to Author:

The authors present a "Technical Note" about an open-source web tool called SpheroScan. As input users could upload (large batches of) spheroid images (brightfield, 2D). The tool delivers two outputs: (1) Prediction Module: creates a file with area and intensity of detected spheroids (CSV), (2) Visualization Module: plots of the corresponding parameters (PNG). Performance was tested on 480 Incucyte images and 423 microscope images with 336 (70 %) and 265 for training, 144 (30 %) and 117 for validation, and 50 images for testing, respectively. The framework is based on Mask R-CNN and Detectron2 library. The performance was tested in the range of 0.5 to 0.95 against manual annotation (VGG Annotator). As evaluation measure they used Intersection over union (IoU), determining the overlap between the predicted and ground truth regions and calculates values of Average Precision (AP) for masking: 0.937 and 0.972 (Test), 0.927 and 0.97 (Validation) as well as AP for bounding box: 0.899 and 0.977 (test) 0.89 and 0.944 (Validation). They show a linear runtime, proofed with different sized datasets (1 s / image) for masking on a 16 core CPU, 64 GB RAM machine. The tool is available on GitHub and claimed to be available as a web tool on spheroscan.onrender.com.General evaluation:The concept of the tool serves some important needs of 3D cell culture-based assays: automated, standardized, high-throughput image analysis. As such, it represents value added for the research field. However, it remains open how high the impact, the reproducibility, and the chances of potential application by other researchers will be. This is due to some significant limitations in accessibility (i.e. non-permanent or non-functional web tool), as well as the (potential) restriction of input data (i.e. brightfield only, not validated with external data) and the limited options for analysis of the metadata (i.e. area and intensity only). The greatest value stems from the possibility to access a web interface, which is easy to use and will ideally be equipped with additional functionalities in the future.Comment 1 (minor):The presented tool uses the Mask R-CNN deep-learning model in their image processing pipeline. Several tools, which perform image segmentation, are based on this or other models are well-established and already implemented in several commercial imaging devices and allow for segmentation of cell containing image areas, e.g. to determine confluency or cell migration in "wound healing assays", mainly optimized for 2D cultures, but also applicable for 2D images of 3D spheroids. The concept of automated image segmentation is thus not novel and only meets the journal's input criterion as "update or adaptation of existing" tools.The state-of-the-art and preliminary work are not sufficiently referenced. Several similar and alternative (open-source) tools are existent and should be mentioned in the manuscript, e.g. (Lacalle et al., 2021; Piccinini et al., 2023; Trossbach et al., 2023), to give only a few examples.Comment 2 (major):The authors claim to present an user-friendly open-source web tool. The python project is available on Github, and on a demo-server (https://spheroscan.onrender.com/) where the web interface can be accessed. Unfortunately the mentioned web tool is not functional, i.e. it is stated on the website: "This is

a demonstration server and the prediction module is not available for use. To utilize the prediction functionality, please run SpheroScan on your local machine.".This is significantly limiting the applicability of the presented tool to users who are able to execute python code on their local hardware. Therefore, the demo server should either present a functional user interface (recommended), or the statement should be removed from the manuscript, which would limit the impact of the submission significantly.Comment 3 (major):The presented algorithm was trained exclusively on internal data of brightfield images from "Incucyte and microscope platforms". Furthermore, two distinct models were generated, working with either Incucyte or microscope images.It remains unclear how the algorithm will perform on external data of prospective users. Given the fact that two distinct models had to be trained for different image sources (i.e. from two different platforms) indicates a limited robustness of the models in this regard. This is clearly a general problem of image processing algorithms, but one that will stand in the way of applicability by external users with certainly other imaging techniques. Since the web tool interface is not functional at this point, the authors will also not be able to evaluate or improve on this after publication. At least one performance test with external data, obtained from an ideally blinded user should be performed, to further elaborate on this.Comment 4 (major):Many assays nowadays use fluorescent labels, for example to calculate cell ratios within 3D arrangements, e.g. for cell viability or the expression of certain proteins. The authors do not state if the algorithm (or future iterations thereof) is or will be able to process multi-channel microscope images of spheroids.This is a significant limitation of the presented work and should at least be mentioned in the corresponding section, respectively. Furthermore, a proof-of-concept test run with fluorescent images could be performed to test the algorithm performance and derive potentially necessary adaptations in future versions.Comment 5 (minor):The output of the tool is a list of detected spheroids with corresponding area (2D) and bright field average intensity within the area.The usability of these two parameters is limited to specific assays, such as the mentioned use case to investigate collagen gel contraction assays. Several other parameters of interest could easily be derived from the metadata, such as roundness, volume estimation (assuming a spheroid shape), or even cell count estimation. This should again be mentioned in the "limitations and considerations" section.

Level of Interest

Please indicate how interesting you found the manuscript: Choose an item.

Quality of Written English

Please indicate the quality of language in the manuscript: Choose an item.

Declaration of Competing Interests

Please complete a declaration of competing interests, considering the following questions:

- Have you in the past five years received reimbursements, fees, funding, or salary from an organisation that may in any way gain or lose financially from the publication of this manuscript, either now or in the future?
- Do you hold any stocks or shares in an organisation that may in any way gain or lose financially from the publication of this manuscript, either now or in the future?
- Do you hold or are you currently applying for any patents relating to the content of the manuscript?
- Have you received reimbursements, fees, funding, or salary from an organization that holds or has applied for patents relating to the content of the manuscript?
- Do you have any other financial competing interests?
- Do you have any non-financial competing interests in relation to this paper?

If you can answer no to all of the above, write 'I declare that I have no competing interests' below. If your reply is yes to any, please give details below.

I declare that I have no competing interests

I agree to the open peer review policy of the journal. I understand that my name will be included on my report to the authors and, if the manuscript is accepted for publication, my named report including any attachments I upload will be posted on the website along with the authors' responses. I agree for my report to be made available under an Open Access Creative Commons CC-BY license (http://creativecommons.org/licenses/by/4.0/). I understand that any comments which I do not wish to be included in my named report can be included as confidential comments to the editors, which will not be published.

Choose an item.

To further support our reviewers, we have joined with Publons, where you can gain additional credit to further highlight your hard work (see: https://publons.com/journal/530/gigascience). On publication of this paper, your review will be automatically added to Publons, you can then choose whether or not to claim your Publons credit. I understand this statement.

Yes Choose an item.
