# Supplementary material for: SpheroScan: a user-friendly deep learning tool for spheroid image analysis
Source: Gigascience. 2023 Oct 27;12:giad082. doi: 10.1093/gigascience/giad082 (PMC10603766; doi:10.1093/gigascience/giad082)
Supplement: giad082_Reviewer_2_Report_Original_Submission — Francesco Pampaloni -- 7/16/2023 Reviewed [file giad082_reviewer_2_report_original_submission.pdf]

Reviewer Report

Title: SpheroScan: A User-Friendly Deep Learning Tool for Spheroid Image Analysis

Version: Original Submission **Date:** 7/16/2023

Reviewer name: Francesco Pampaloni

Reviewer Comments to Author:

This study represents a significant contribution to the field of screening and analysis of three-dimensional cell cultures. The demand for reliable and user-friendly image processing tools to extract quantitative data from a large number of spheroids or other types of three-dimensional tissue models is substantial. The authors of this manuscript have developed a tool that aims to address this need by providing a straightforward method to extract the projected area and intensity of individual cellular spheroids imaged with bright-field microscopy. The tool is compatible with "Incucyte" microscopes or any other automated microscope capable of imaging multiple specimens, typically found in high-density multiwell plates.An admirable aspect of this work is the authors' decision to make all the code and pipeline openly available on Github. This openness allows other scientists to test and validate the code, promoting transparency and collaboration in the scientific community. However, several improvements should be made to the manuscript prior to publication.One important aspect that the authors should address in the manuscript is the suitability, rationale, and extent of using a neural network-based segmentation approach for the specific analysis described in the manuscriptâ€”segmentation of single bright-field images of spheroids. While neural networks are anticipated to play an increasingly important role in microscopy data segmentation in the coming years, they are not a universal solution. Although there may be segmentation tasks that are challenging to accomplish with traditional approaches, where neural networks can be highly effective, other segmentation tasks can be successfully performed using conventional strategies. For example, in our research group, we were able to reliably segment densely populated bright-field images containing numerous organoids in a single field of view using a pipeline based on the ImageJ plugin MorphoLibJ (see references: https://doi.org/10.1093/bioinformatics/btw413 and https://doi.org/10.1186/s12915-021-00958-w). Therefore, it would be informative and valuable for readers if the authors compared the results obtained from the neural network with those achieved by employing simple thresholding techniques (such as Otsu or Watershed) on the same dataset, as demonstrated in a similar study (reference: https://doi.org/10.1038/s41598-021-94217-1, Figure 5).Furthermore, to address the limitations of the model, the authors should provide specific examples (preferably in the supplementary material due to space constraints) of incorrect segmentations or artifacts that arise from applying the neural network to the data. For instance, it would be beneficial to explore scenarios where spheroids are surrounded by cellular debris or when multiple spheroids are present in the field of view. These real-life situations are common and it is important to provide insights into potential challenges that may arise when the images of the spheroids are not pristine.

Level of Interest

Please indicate how interesting you found the manuscript: Choose an item.

Quality of Written English

Please indicate the quality of language in the manuscript: Choose an item.

Declaration of Competing Interests

Please complete a declaration of competing interests, considering the following questions:

- Have you in the past five years received reimbursements, fees, funding, or salary from an organisation that may in any way gain or lose financially from the publication of this manuscript, either now or in the future?
- Do you hold any stocks or shares in an organisation that may in any way gain or lose financially from the publication of this manuscript, either now or in the future?
- Do you hold or are you currently applying for any patents relating to the content of the manuscript?
- Have you received reimbursements, fees, funding, or salary from an organization that holds or has applied for patents relating to the content of the manuscript?
- Do you have any other financial competing interests?
- Do you have any non-financial competing interests in relation to this paper?

If you can answer no to all of the above, write 'I declare that I have no competing interests' below. If your reply is yes to any, please give details below.

No competing interests

I agree to the open peer review policy of the journal. I understand that my name will be included on my report to the authors and, if the manuscript is accepted for publication, my named report including any attachments I upload will be posted on the website along with the authors' responses. I agree for my report to be made available under an Open Access Creative Commons CC-BY license (http://creativecommons.org/licenses/by/4.0/). I understand that any comments which I do not wish to be included in my named report can be included as confidential comments to the editors, which will not be published.

Choose an item.

To further support our reviewers, we have joined with Publons, where you can gain additional credit to further highlight your hard work (see: https://publons.com/journal/530/gigascience). On publication of this paper, your review will be automatically added to Publons, you can then choose whether or not to claim your Publons credit. I understand this statement.

Yes Choose an item.
